# Synthesis and evaluation of a large library of nitroxoline derivatives as pancreatic cancer antiproliferative agents

**DOI:** 10.1080/14756366.2020.1780228

**Published:** 2020-06-26

**Authors:** Serena Veschi, Simone Carradori, Laura De Lellis, Rosalba Florio, Davide Brocco, Daniela Secci, Paolo Guglielmi, Mattia Spano, Anatoly P. Sobolev, Alessandro Cama

**Affiliations:** a Department of Pharmacy, “G. d’Annunzio” University of Chieti-Pescara, Chieti, Italy; b Dipartimento di Chimica e Tecnologie del Farmaco, Sapienza Università di Roma, Rome, Italy; c Istituto per i Sistemi Biologici, Laboratorio di Risonanza Magnetica “Segre-Capitani”, CNR, Monterotondo (Rome), Italy

**Keywords:** Nitroxoline derivatives, drug repurposing, pancreatic cancer, Erlotinib, clonogenicity, 4-nitro(thio)phenyl

## Abstract

Pancreatic cancer (PC) is one of the deadliest carcinomas and in most cases, which are diagnosed with locally advanced or metastatic disease, current therapeutic options are highly unsatisfactory. Based on the anti-proliferative effects shown by nitroxoline, an old urinary antibacterial agent, we explored a large library of newly synthesised derivatives to unravel the importance of the OH moiety and pyridine ring of the parent compound. The new derivatives showed a valuable anti-proliferative effect and some displayed a greater effect as compared to nitroxoline against three pancreatic cancer cell lines with different genetic profiles. In particular, *in silico* pharmacokinetic data, clonogenicity assays and selectivity indexes of the most promising compounds showed several advantages for such derivatives, as compared to nitroxoline. Moreover, some of these novel compounds had stronger effects on cell viability and/or clonogenic capacity in PC cells as compared to erlotinib, a targeted agent approved for PC treatment.

## 1. Introduction

Pancreatic cancer (PC) is one of the deadliest neoplasms, with a survival rate at 5 years of less than 6%^1^. Incidence and mortality rates for PC are increasing and its unfavourable prognosis is due to poor response to current therapies^2^^,^^3^. Thus, it is of paramount importance to find novel and more effective therapeutic approaches. In this respect, it has been reported that several non-anticancer agents approved for the treatment of different human diseases may have anticancer properties^4^. Our recent studies provided the first evidence that the non-antitumor drug nitroxoline has antitumor effects on PC cells^5^^,^^6^. Previously, this antibiotic showed antitumour effects in preclinical cancer models including xenograft and genetically modified mice models of glioblastoma, myeloma, fibrosarcoma as well as bladder, prostate, kidney and breast cancers^7–13^. In line with the results obtained in other tumours, we showed that nitroxoline significantly reduces cell viability, affects cell cycle, promotes apoptosis and markedly decreases clonogenic activity in PC cells^5^. Moreover, using integrative proteomic and functional approaches, we evaluated nitroxoline effects on protein expression in PC cells, showing that this drug affects several biological pathways and oncogenic proteins, previously unknown to play a role in nitroxoline anticancer activity^6^. Notably, our study revealed a pleiotropic mechanism of nitroxoline anticancer action that involves processes playing a crucial role in growth, migration, invasion and cell death, ROS production and DNA damage^6^. Remarkably, this drug induced a previously unknown deregulation of molecules that are involved in cell bioenergetics, leading to mitochondrial depolarisation^6^. Moreover, recent evidence suggests that, besides its direct action on tumour cells, nitroxoline may activate antitumour immune response, which is recognised to be crucial in novel and more effective anticancer strategies^14^^,^^15^. Indeed, the immune system plays a crucial role in long-term cancer response to chemotherapy^16^. Overall, these data indicate a marked antitumour effect of nitroxoline on different cancers including PC, suggesting that this drug is a promising candidate for repositioning in the treatment of PC.

For these reasons, nitroxoline structure has prompted the Medicinal chemists to explore the structural requirements suitable to improve antitumor effects. In previous papers, taking advantage of the reactivity of the quinoline nucleus, most authors studied the introduction of halogens and additional side chains as well as modifications of the nitro group^17^^,^^18^. A limitation of most previous efforts was that they were directed towards the cathepsin B inhibitory activity, disregarding the other mechanisms of action demonstrated for nitroxoline as an anticancer compound so far. The aim of our study was the hitherto unexplored modification of the OH group. This could lead to a strong alteration of the chemical–physical characteristics of this 8-hydroxyquinoline. Indeed, this functional group endowed the chemical structure with a discrete acidity (reinforced by the *p*-NO_2_ moiety) and chelating ability. Furthermore, this phenolic compound is characterised by proton and electron-donating capacity and, as a consequence, antioxidant properties which can occur during several important biological processes^19^.

Starting from these premises and pursuing our efforts towards the discovery of new anti-proliferative heterocyclic agents^20–22^, we functionalised the OH of nitroxoline in order to obtain a large library of ether analogues introducing linear and branched (C_2_-C_15_), saturated and unsaturated alkyl chains, and substituted and unsubstituted aryl and bicyclic rings. Moreover, we inserted a carbonyl spacer between the benzylic methylene and the aryl ring to evaluate the impact of this elongation. Aliphatic ethers were also decorated with COOH, CN, and COOEt to further explore the chemical space around these positions. Benzylic ethers were characterised by the presence of electron-donating (methyl, methoxyl, thiomethyl) and electron-withdrawing (halogens, nitro, cyano, trifluoromethyl) substituents in *ortho*, *meta* and *para* positions. Polysubstitutions were also provided. Lastly, to better understand the importance of the quinoline nucleus, we aimed at simplifying the nitroxoline structure to 4-nitro(thio)phenol. On these two scaffolds, we added the same substituents that resulted to be more active in the first series giving the possibility to extrapolate robust structure-activity relationships (SARs).

All the gathered compounds have been first tested in a one-point concentration screening against three human PC cell lines to assess the best-in-class compounds for further cell-based experiments. Specifically, we selected AsPC-1, Capan-2 and BxPC-3 pancreatic cancer cell lines that display different genetic profiles: AsPC-1 and Capan-2 are *KRAS* mutated, while BxPC-3 and AsPC-1 carry *p53* mutations.

## 2. Chemistry

**Figure 1.**
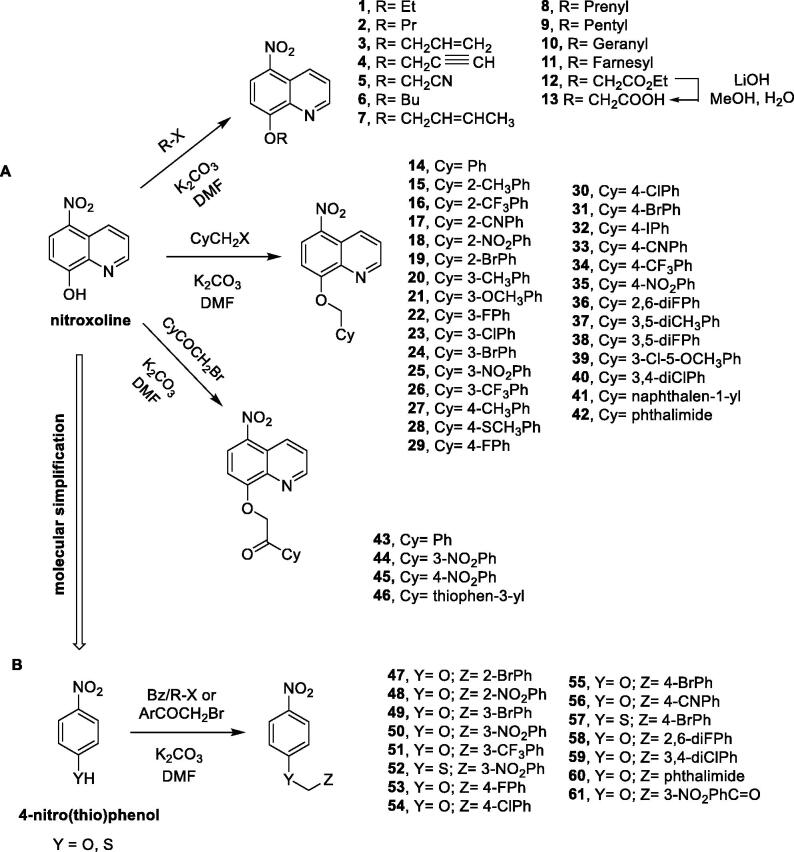
Synthesis of compounds **1**–**61**.

For the synthesis of compounds **1**–**61,** we followed the synthetic approaches outlined in Figure 1. Derivatives **1**–**12**, **14**–**46** (Figure 1(A)) have been easily synthesised by reacting nitroxoline with the proper alkyl/benzyl bromides or α-bromoacetophenones; these reactions were performed in *N*,*N*’-dimethylformamide (DMF), in the presence of potassium carbonate (K_2_CO_3_) and under nitrogen (N_2_) atmosphere. In addition, compound **12** was hydrolysed in mild conditions using lithium hydroxide (LiOH) in a mixture of water and methanol (in the ratio 50:50*, v:v*) at room temperature, to provide the carboxylic acid derivative **13**. For compounds **47**–**61**, the same reactions involving 4-nitrophenol or 4-nitrothiophenol were performed (Figure 1(B)). The structures were confirmed by spectral studies (^1^H/^13^C/^19^F NMR), whereas the purity of these compounds was confirmed by combustion analysis, TLC parameters, crystallographic studies (for compound **16**) and melting point evaluation. *In silico* analysis of the most active compounds was performed by using the online free software SwissADME, a web tool that allows to appraise pharmacokinetics, as well as drug-likeness (the probability to be an oral drug) and medicinal chemistry friendliness (PAINS) of small molecules^23^. Target prediction was attempted taking advantage of the SwissTargetPrediction web tool^24^.

## 3. X-ray diffraction analysis

**Figure 2.**
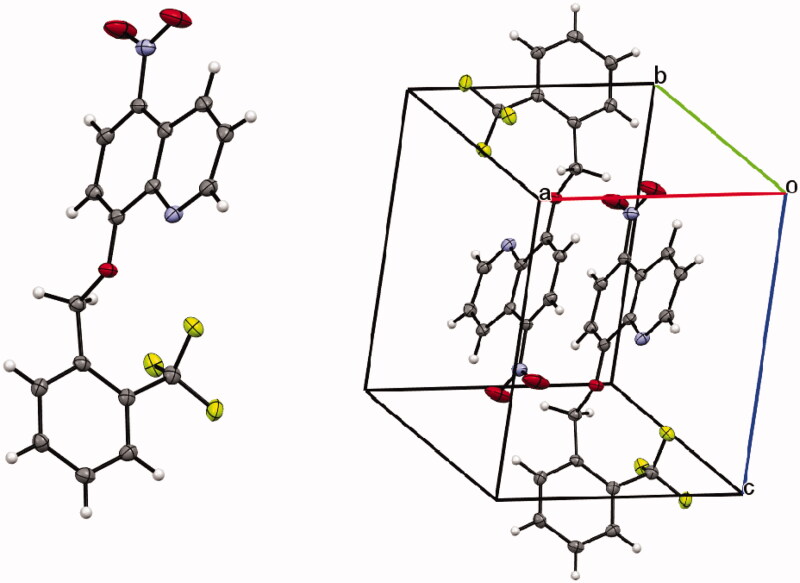
Crystal structure of nitroxoline-based compound **16**.

Crystals of compound **16** (Figure 2) were obtained by slow evaporation from an ethyl acetate/*n*-hexane mixture. Information about the crystal data, experimental collection conditions and refinement as well as the structural geometric parameters are available in the Cambridge Crystallographic Data Centre in CIF format (CCDC 2001211).

## 4. Materials and methods

### 4.1. Chemistry

Unless otherwise indicated, all reactions were carried out under a positive pressure of nitrogen in washed and oven-dried glassware. All the solvents and reagents were directly used as supplied by Sigma-Aldrich (Milan, Italy) without further purification unless otherwise noted. Where mixtures of solvents are specified, the stated ratios are volume:volume. All melting points were measured on a Stuart^®^ melting point apparatus SMP1 and are uncorrected (temperatures are reported in °C). ^1^H and ^13 ^C NMR spectra were recorded at 300 MHz and 75 MHz (Varian Mercury spectrometer), or at 400.13 MHz and 101.03 MHz on a Bruker spectrometer, using CDCl_3_ and DMSO-*d_6_*, as the solvents at room temperature. ^19 ^F spectra were recorded on a Bruker AVANCE 600 spectrometer at 564.7 MHz, using CDCl_3_ as solvent. All the compounds were analysed with a final concentration of ∼25 mg/mL. ^1^H and ^13 ^C chemical shifts are expressed as *δ* units (parts per millions) relative to the solvent signal (DMSO-*d*_6_: ^1^H 2.50 and ^13 ^C 39.5; CDCl_3_: ^1^H 7.26 and ^13 ^C 77.4), whereas ^19 ^F chemical shifts are expressed as *δ* units relative to an external standard (CF_3_COOH, *δ* − 76.55 ppm). ^1^H spectra are described as follows: *δ*_H_ (spectrometer frequency, solvent): chemical shift/ppm (multiplicity, *J*-coupling constant(s), number of protons, assignment). ^13 ^C spectra are described as follows: *δ*_C_ (spectrometer frequency, solvent): chemical shift/ppm (assignment) and are fully proton decoupled. ^19 ^F spectra are described as follows: *δ*_F_ (spectrometer frequency, solvent): chemical shift/ppm (multiplicity, *J*-coupling constant(s), number of fluorine, assignment). Multiplets are abbreviated as follows: br – broad; s – singlet; d – doublet; t – triplet; q – quartette; td – triplet of doublets; m – multiplet. Coupling constants *J* are valued in Hertz.

The processing and analyses of the NMR data were carried out with MestreNova. Elemental analyses for C, H, and N were recorded on a Perkin-Elmer 240 B microanalyzer obtaining analytical results within ± 0.4% of the theoretical values for the final compounds. Preparative chromatography was carried out employing Sigma-Aldrich^®^ silica gel (high purity grade, pore size 60 Å, 230–400 mesh particle size). All the purifications and reactions were carried out by TLC performed on 0.2 mm thick silica gel-aluminium backed plates (60 F254, Merck). Spot visualisation was performed under short- and long-wavelength (254 and 365 nm, respectively) ultra-violet irradiation. Where given, systematic compound names and ClogP values are those generated by ChemBioDraw Ultra 14.0 following IUPAC conventions.

### 4.2. Synthesis of nitroxoline derivatives

#### 4.2.1. General procedure for the synthesis of compounds 1–12 and 14–46

Freshly ground potassium carbonate (K_2_CO_3_, 1.1 equiv.) was added to a stirring solution of nitroxoline (1 equiv.) in DMF (20 ml). The yellow suspension was stirred for 30 min at room temperature; then, the proper (substituted)benzyl, diaryl or alkyl bromide or α-bromoacetophenone (1.5 equiv.) was added and the reaction stirred until disappearance of the starting reagents, as detected by thin layer chromatography (TLC). Then, the mixture was poured into ice-cold water (150 ml) and partitioned with dichloromethane (DCM, 3 × 40 ml). The organics were separated, reunited and added with anhydrous sodium sulphate (Na_2_SO_4_) to remove water. The salt was filtered and washed two times with 5 ml of dry DCM. The organic phase was evaporated *in vacuo* to afford the crude extract containing the target molecule that was recovered through column chromatography, employing silica gel (SiO_2_) and proper mixtures of *n*-hexane/ethyl acetate.

#### 4.2.2. Synthesis of compound 13

Lithium hydroxide (1.1 equiv.) dissolved in 15 ml of water was added dropwise to a stirring solution of ethyl 2-((5-nitroquinolin-8-yl)oxy)acetate (**12**, 1.0 equiv.) in 30 ml of methanol. The reaction was stirred at room temperature for 24 h; then, the mixture was concentrated *in vacuo* to remove methanol and quenched with 3 N HCl (15 ml). The precipitate was collected by filtration and washed with *n*-hexane to give the title compound **13**, without further purification requirements.

#### 4.2.3. General procedure for the synthesis of compounds 47–61

Freshly ground potassium carbonate (K_2_CO_3_, 1.1 equiv.) was added to a stirring solution of 4-nitro(thio)phenol (1 equiv.) in DMF (10 ml). The yellow suspension was stirred for 30 min at room temperature; then, the proper (substituted)benzyl, diaryl and alkyl bromide or α-bromoacetophenone (1.5 equiv.) was added and the reaction stirred until disappearance of the starting reagents, as detected by thin layer chromatography. Then, the mixture was poured into ice-cold water (100 ml) and extracted with dichloromethane (DCM, 3 × 25 ml). The organics were reunited and added with anhydrous sodium sulphate to remove water. The salt was filtered and washed three times with 5 ml of dry DCM. The organic phase was evaporated *in vacuo* to afford the crude extract containing the target molecule that was recovered through column chromatography, employing silica gel and proper mixtures of *n*-hexane/ethyl acetate.

### 4.3. Characterisation data for nitroxoline and 4-nitro(thio)phenol derivatives

*8-ethoxy-5-nitroquinoline* (**1**). Yellow solid, 66% yield, ClogP 2.71, mp = 126–128 °C. ^1^H NMR (400 MHz, CDCl_3_): *δ* 1.68–1.71 (*t*, *J* = 7.0 Hz, 3H, CH_3_), 4.44–4.49 (*q*, *J* = 7.0 Hz, 2H, OCH_2_), 7.08–7.10 (d, *J* = 8.9 Hz, 1H, ArH), 7.69–7.72 (*m*, 1H, ArH), 8.53–8.55 (d, *J* = 8.9 Hz, 1H, ArH), 9.07–9.08 (*m*, 1H, ArH), 9.24–9.27 (*m*, 1H, ArH); ^13 ^C NMR (100 MHz, CDCl_3_): *δ* 14.4 (CH_3_), 65.6 (CH_2_), 106.0 (Ar), 123.1 (Ar), 124.5 (Ar), 127.7 (Ar), 132.7 (Ar), 137.4 (Ar), 139.3 (Ar), 150.1 (Ar), 160.2 (Ar). Anal. calcd for C_11_H_10_N_2_O_3_: C, 60.55; H, 4.62; N, 12.84, found: C, 60.51; H, 4.60; N, 12.82.

*5-nitro-8-propoxyquinoline* (**2**). Yellow solid, 72% yield, ClogP 3.24, mp = 110–112 °C. ^1^H NMR (300 MHz, CDCl_3_): *δ* 1.08–1.13 (*t*, *J* = 7.6 Hz, 3H, CH_3_), 2.00–2.12 (*m*, 2H, CH_2_), 4.26–4.31 (*t*, *J* = 7.2 Hz, 2H, OCH_2_), 7.02–7.05 (d, *J* = 9.0 Hz, 1H, ArH), 7.63–7.68 (*m*, 1H, ArH), 8.47–8.50 (d, *J* = 8.7 Hz, 1H, ArH), 9.00–9.03 (*m*, 1H, ArH), 9.18–9.21 (*m*, 1H, ArH); ^13 ^C NMR (75 MHz, CDCl_3_): *δ* 10.4 (CH_3_), 22.0 (CH_2_), 71.5 (OCH_2_), 123.1 (Ar), 124.5 (Ar), 127.7 (Ar), 132.7 (Ar), 106.1 (Ar), 137.3 (Ar), 139.1 (Ar), 150.0 (Ar), 160.3 (Ar). Anal. calcd for C_12_H_12_N_2_O_3_: C, 62.06; H, 5.21; N, 12.06, found: C, 62.00; H, 5.19; N, 12.00.

*8-(allyloxy)-5-nitroquinoline* (**3**). Brown solid, 81% yield, ClogP 2.96, mp = 102–103 °C. ^1^H NMR (300 MHz, CDCl_3_): *δ* 4.97–4.99 (d, *J* = 1.2 Hz, 2H, OCH_2_), 5.39–5.54 (*m*, 2H, =CH_2_), 6.12–6.25 (*m*, 1H, =CH), 7.07–7.10 (d, *J* = 9.0 Hz, 1H, ArH), 7.68–7.72 (*m*, 1H, ArH), 8.49–8.52 (d, *J* = 8.7 Hz, 1H, ArH), 9.05–9.07 (*m*, 1H, ArH), 9.22–9.26 (*m*, 1H, ArH); ^13 ^C NMR (75 MHz, CDCl_3_): *δ* 70.6 (CH_2_), 106.9 (Ar), 119.7 (CH_2_=), 123.1 (Ar), 124.6 (Ar), 127.5 (Ar), 131.5 (Ar), 133.0 (CH=), 150.0 (Ar), 159.6 (Ar). Anal. calcd for C_12_H_10_N_2_O_3_: C, 62.61; H, 4.38; N, 12.17, found: C, 62.66; H, 4.40; N, 12.21.

*5-nitro-8-(prop-2-yn-1-yloxy)quinoline* (**4**). Brown solid, 68% yield, CLogP 2.60, mp = 157–158 °C. ^1^H NMR (300 MHz, CDCl_3_): *δ* 2.62–2.63 (bs, 1H, C≡CH), 5.14–5.15 (bs, 2H, OCH_2_), 7.31–7.34 (d, *J* = 8.7 Hz, 1H, ArH), 7.72–7.77 (*m*, 1H, ArH), 8.55–8.58 (d, *J* = 8.7 Hz, 1H, ArH), 9.07–9.09 (bs, 1H, ArH), 9.26–9.29 (d, *J* = 9.3 Hz, 1H, ArH); ^13 ^C NMR (75 MHz, CDCl_3_): *δ* 57.2 (OCH_2_), 76.6 (HC≡, overlapping with the solvent signals), 77.8 (C≡), 107.9 (Ar), 123.1 (Ar), 124.7 (Ar), 127.5 (Ar), 134.0 (Ar), 138.2 (Ar), 149.7 (Ar), 157.7 (Ar). Anal. calcd for C_12_H_8_N_2_O_3_: C, 63.16; H, 3.53; N, 12.28, found: C, 63.06; H, 3.50; N, 12.20.

*2-((5-nitroquinolin-8-yl)oxy)acetonitrile* (**5**). Orange solid, 65% yield, ClogP 1.22, mp = 198–200 °C [Lit. mp = 194–197 °C]. Characterisation data are in agreement with those reported in the literature^18^.

*8-butoxy-5-nitroquinoline* (**6**). Brown solid, 78% yield, ClogP 3.77, mp = 104–106 °C. ^1^H NMR (300 MHz, CDCl_3_): *δ* 1.00–1.05 (*t*, *J* = 7.5 Hz, 3H, CH_3_), 1.52–1.65 (*m*, 2H, CH_2_), 2.00–2.10 (*m*, 2H, CH_2_), 4.34–4.39 (*t*, *J* = 7.2 Hz, 2H, OCH_2_), 7.07–7.10 (d, *J* = 9.0 Hz, 1H, ArH), 7.68–7.72 (*m*, 1H, ArH), 8.52–8.55 (d, *J* = 9.0 Hz, 1H, ArH), 9.06–9.08 (*m*, 1H, ArH), 9.25–9.28 (*m*, 1H, ArH); ^13 ^C NMR (75 MHz, CDCl_3_): *δ* 13.8 (CH_3_), 19.2 (CH_2_), 30.6 (CH_2_), 70.0 (OCH_2_), 106.4 (Ar), 123.2 (Ar), 124.5 (Ar), 128.0 (Ar), 133.5 (Ar), 137.2 (Ar), 149.7 (Ar), 160.0 (Ar). Anal. calcd for C_13_H_14_N_2_O_3_: C, 63.40; H, 5.73; N, 11.38, found: C, 63.20; H, 5.63; N, 11.12.

*(E)-8-(but-2-en-1-yloxy)-5-nitroquinoline* (**7**). This compound exists in a mixture of *E*/*Z* isomers with the following ratio (4.4/1) as extrapolated by their OCH_2_ signal integration in the ^1^H NMR spectrum (peaks listed only for the major isomer). Brown solid; 79% yield, ClogP 3.49, mp = 87–89 °C; ^1^H NMR (300 MHz, CDCl_3_): *δ* 1.70–1.72 (d, *J* = 6.6 Hz, 3H, CH_3_), 4.79–4.81 (d, *J* = 6.4 Hz, 2H, OCH_2_), 5.76–5.96 (*m*, 2H, 2 x = CH), 6.99–7.02 (d, *J* = 9.0 Hz, 1H, ArH), 7.57–7.61 (*m*, 1H, ArH), 8.40–8.43 (d, *J* = 9.0 Hz, 1H, ArH), 8.94–8.96 (*m*, 1H, ArH), 9.10–9.13 (*m*, 1H, ArH); ^13 ^C NMR (75 MHz, CDCl_3_): *δ* 17.9 (CH_3_), 70.5 (OCH_2_), 106.5 (Ar), 123.0 (Ar), 124.1 (Ar), 124.4 (CH=), 127.5 (Ar), 129.9 (CH=), 132.5 (Ar), 137.3 (Ar), 139.3 (Ar), 150.1 (Ar), 159.9 (Ar). Anal. calcd for C_13_H_12_N_2_O_3_: C, 63.93; H, 4.95; N, 11.47, found: C, 63.69; H, 4.80; N, 11.22.

*8-((3-methylbut-2-en-1-yl)oxy)-5-nitroquinoline* (**8**). Yellow solid, 82% yield, ClogP 3.88, mp = 98–99 °C. ^1^H NMR (300 MHz, CDCl_3_): *δ* 1.81 (*s*, 6H, 2 x CH_3_), 4.94–4.96 (d, *J* = 6.3 Hz, 2H, OCH_2_), 5.60–5.64 (*m*, 1H, =CH), 7.05–7.08 (d, *J* = 8.7 Hz, 1H, ArH), 7.67–7.13 (*m*, 1H, ArH), 8.51–8.54 (d, *J* = 8.7 Hz, 1H, ArH), 9.05–9.07 (*m*, 1H, ArH), 9.24–9.27 (*m*, 1H, ArH); ^13 ^C NMR (75 MHz, CDCl_3_): *δ* 18.4 (CH_3_), 25.9 (CH_3_), 66.9 (OCH_2_), 106.6 (Ar), 118.3 (CH=), 123.1 (Ar), 124.5 (Ar), 127.8 (Ar), 133.1 (Ar), 139.5 (C), 149.9 (Ar), 160.0 (Ar). Anal. calcd for C_14_H_14_N_2_O_3_: C, 65.11; H, 5.46; N, 10.85, found: C, 65.33; H, 5.60; N, 11.00.

*5-nitro-8-(pentyloxy)quinoline* (**9**). Brown sticky oil, 83% yield, ClogP 4.30.^1^H NMR (300 MHz, CDCl_3_): *δ* 0.90–0-94 (*t*, *J* = 6.9 Hz, 3H, CH_3_), 1.39–1.53 (*m*, 4H, 2 × CH_2_), 2.01–2.06 (*m*, 2H, CH_2_), 4.30–4.34 (*t*, *J* = 7.2 Hz, 2H, OCH_2_), 7.02–7.05 (d, *J* = 9.6 Hz, 1H, ArH), 7.63–7.67 (*m*, 1H, ArH), 8.47–8.50 (d, *J* = 8.7 Hz, 1H, ArH), 9.01–9.02 (bd, *J* = 2.7 Hz, 1H, ArH), 9.18–9.21 (d, *J* = 8.7 Hz, 1H, ArH); ^13 ^C NMR (75 MHz, CDCl_3_): *δ* 14.0 (CH_3_), 22.4 (CH_2_), 28.0 (CH_2_), 28.4 (CH_2_), 70.1 (OCH_2_), 106.1 (Ar), 123.1 (Ar), 124.5 (Ar), 127.7 (Ar), 132.7 (Ar), 137.2 (Ar), 139.2 (Ar), 150.1 (Ar), 160.4 (Ar). Anal. calcd for C_14_H_16_N_2_O_3_: C, 64.60; H, 6.20; N, 10.76, found: C, 64.39; H, 6.09; N, 10.52.

*(E)-8-((3,7-dimethylocta-2,6-dien-1-yl)oxy)-5-nitroquinoline* (**10**). Brown sticky oil, 91% yield, ClogP 5.92. ^1^H NMR (300 MHz, CDCl_3_): *δ* 1.54 (*s*, 3H, CH_3_), 1.60 (*s*, 3H, CH_3_), 1.78 (*s*, 3H, CH_3_), 2.07 (bs, 4H, 2 × CH_2_), 4.94–5.02 (*m*, 3H, OCH_2_ + =CH), 5.55–5.59 (bs, 1H, =CH), 7.00–7.03 (d, *J* = 8.7 Hz, 1H, ArH), 7.61–7.66 (*m*, 1H, ArH), 8.45–8.48 (d, *J* = 8.7 Hz, 1H, ArH), 8.99–9.00 (d, *J* = 3.6 Hz, 1H, ArH), 9.17–9.20 (d, *J* = 8.7 Hz, 1H, ArH); ^13 ^C NMR (75 MHz, CDCl_3_): *δ* 16.9 (CH_3_), 17.7 (CH_3_), 25.6 (CH_3_), 26.1 (CH_2_), 39.5 (CH_2_), 67.0 (OCH_2_), 106.5 (Ar), 118.2 (Ar), 123.1 (CH=), 123.5 (Ar), 124.4 (Ar), 127.6 (Ar), 131.9 (CH=), 132.7 (C), 137.2 (Ar), 139.3 (C), 142.3 (Ar), 150.0 (Ar), 160.1 (Ar). Anal. calcd for C_19_H_22_N_2_O_3_: C, 69.92; H, 6.79; N, 8.58, found: C, 69.45; H, 6.58; N, 8.22.

*5-nitro-8-(((3,7,11-trimethyldodeca-2,6,10-trien-1-yl)oxy)quinoline* (**11**). The compound exists as a mixture of *E*/*Z* isomers (peaks listed only for the major isomer). Dark brown sticky oil, 77% yield, ClogP 7.95. ^1^H NMR (300 MHz, CDCl_3_): *δ* 1.50 (*s*, 3H, CH_3_), 1.52 (*s*, 3H, CH_3_), 1.59 (*s*, 3H, CH_3_), 1.77 (*s*, 3H, CH_3_), 1.87–2.07 (*m*, 8H, 4 × CH_2_), 4.91–5.02 (*m*, 4H, OCH_2_ + 2 x = CH), 5.56 (*s,* 1H, CH=), 6.97–7.00 (d, *J* = 8.7 Hz, 1H, ArH), 7.57–7.60 (bs, 1H, ArH), 8.41–8.44 (d, *J* = 8.7 Hz, 1H, ArH), 8.95 (*s*, 1H, ArH), 9.12–9.15 (d, *J* = 8.7 Hz, 1H, ArH); ^13 ^C NMR (75 MHz, CDCl_3_): *δ* 16.0 (CH_3_), 16.8 (CH_3_), 17.6 (CH_3_), 25.4 (CH_3_), 26.0 (CH_2_), 26.6 (CH_2_), 39.2 (CH_2_), 39.6 (CH_2_), 66.7 (OCH_2_), 106.3 (Ar), 118.0 (CH=), 123.0 (Ar), 123.4 (CH=), 124.0 (Ar), 124.3 (CH=), 127.4 (C), 131.2 (Ar), 132.4 (C), 135.5 (C), 137.2 (Ar), 139.4 (C), 142.3 (Ar), 150.0 (Ar), 160.1 (Ar). Anal. calcd for C_24_H_30_N_2_O_3_: C, 73.07; H, 7.67; N, 7.10, found: C, 72.80; H, 7.49; N, 6.88.

*ethyl 2-((5-nitroquinolin-8-yl)oxy)acetate* (**12**). Ochre solid, 88% yield, ClogP 2.33, mp = 178–179 °C [Lit. mp = 174–175 °C]. Characterisation data are in agreement with those reported in the literature^18^.

*2-((5-nitroquinolin-8-yl)oxy)acetic acid* (**13**). Yellow solid; 81% yield, ClogP 1.47, mp = 205–207 °C [Lit. mp = 205–210 °C]. Characterisation data are in agreement with those reported in the literature^18^.

*8-(benzyloxy)-5-nitroquinoline* (**14**). Dark red sticky solid, 82% yield, CloP 3.95. ^1^H NMR (300 MHz, CDCl_3_): *δ* 5.48 (bs, 2H, OCH_2_), 6.97–7.01 (*m*, 1H, ArH), 7.26–7.37 (*m*, 3H, 3 × ArH), 7.45–7.48 (*m*, 2H, 2 × ArH), 7.60–7.64 (*m*, 1H, ArH), 8.32–8.37 (*m*, 1H, ArH), 8.99–9.01 (bs, 1H, ArH), 9.09–9.15 (*m*, 1H, ArH); ^13 ^C NMR (75 MHz, CDCl_3_): *δ* 71.4 (OCH_2_), 107.2 (Ar), 123.0 (Ar), 124.5 (Ar), 127.1 (Ar), 127.3 (Ar), 128.4 (Ar), 128.9 (Ar), 132.4 (Ar), 135.3 (Ar), 137.6 (Ar), 139.5 (Ar), 150.2 (Ar), 159.7 (Ar). Anal. calcd for C_16_H_12_N_2_O_3_: C, 68.56; H, 4.32; N, 9.99, found: C, 68.82; H, 4.41; N, 10.16.

*8-((2-methylbenzyl)oxy)-5-nitroquinoline* (**15**). Yellow solid, 93% yield, ClogP 4.40, mp = 126–128 °C. ^1^H NMR (300 MHz, CDCl_3_): *δ* 2.45 (*s*, 3H, CH_3_), 5.50 (*s*, 2H, OCH_2_), 7.06–7.10 (d, *J* = 9.0 Hz, 1H, ArH), 7.19–7.26 (*m*, 3H, ArH), 7.44–7.46 (d, *J* = 6.9 Hz, 1H, ArH), 7.68–7.72 (*m*, 1H, ArH), 8.46–8.49 (d, *J* = 8.7 Hz, 1H, ArH), 9.05–9.06 (bs, 1H, ArH), 9.22–9.25 (d, *J* = 8.7 Hz, 1H ArH); ^13 ^C NMR (75 MHz, CDCl_3_): *δ* 19.1 (CH_3_), 70.2 (OCH_2_), 107.2 (Ar), 123.2 (Ar), 124.6 (Ar), 126.2 (Ar), 127.4 (Ar), 128.2 (Ar), 128.6 (Ar), 130.7 (Ar), 132.8 (Ar), 133.0 (Ar), 136.4 (Ar), 137.8 (Ar), 139.4 (Ar), 150.2 (Ar), 159.8 (Ar). Anal. calcd for C_17_H_14_N_2_O_3_: C, 69.38; H, 4.79; N, 9.52, found: C, 69.01; H, 4.59; N, 9.27.

*5-nitro-8-((2-(trifluoromethyl)benzyl)oxy)quinoline* (**16**). Yellow solid, 82% yield, ClogP 4.83, mp = 145–147 °C. ^1^H NMR (300 MHz, CDCl_3_): *δ* 5.66 (*s*, 2H, OCH_2_), 6.93–6.96 (*m*, 1H, ArH), 7.36–7.41 (*t, J* = 7.5 Hz, 1H, ArH), 7.47–7.52 (*t*, *J* = 7.5 Hz, 1H, ArH), 7.63–7.69 (*m*, 2H, ArH), 7.75–7.77 (d, *J* = 7.5 Hz, 1H, ArH), 8.35–8.38 (*m*, 1H, ArH), 9.02–9.04 (*m*, 1H, ArH), 9.12–9.16 (*m*, 1H, ArH); ^13 ^C NMR (100 MHz, CDCl_3_): *δ* 67.3 (OCH_2_), 107.2 (Ar), 123.0 (d, *J*_C-F_ = 2.2 Hz, Ar), 124.3 (d, ^1^*J*_C-F_ = 280.3 Hz, CF_3_), 124.6 (Ar), 126.0 (Ar), 126.1 (Ar), 127.3 (Ar), 127.9 (Ar), 128.2 (Ar), 132.6 (d, ^3^*J*_C-F_ = 11.5 Hz, Ar), 132.8 (Ar), 133.9 (Ar), 138.0 (Ar), 139.3 (Ar), 150.3 (Ar), 159.0 (Ar); ^19 ^F NMR (564.7 MHz CDCl_3_): *δ* − 58.93 (s, 3 F, ArCF_3_). Anal. calcd for C_17_H_11_F_3_N_2_O_3_: C, 58.63; H, 3.18; N, 8.04, found: C, 58.40; H, 3.02; N, 7.90.

*2-(((5-nitroquinolin-8-yl)oxy)methyl)benzonitrile* (**17**). Yellowish solid, 82% yield, ClogP 3.52, mp = 201–203 °C. ^1^H NMR (300 MHz, CDCl_3_): *δ* 5.68 (*s*, 2H, OCH_2_), 7.09–7.13 (*m*, 1H, ArH), 7.44–7.49 (*t*, *J* = 7.5 Hz, 1H, ArH), 7.61–7.81 (*m*, 4H, 4 × ArH), 8.44–8.48 (*m*, 1H, ArH), 9.05–9.07 (*m*, 1H, ArH), 9.18–9.22 (*m*, 1H, ArH); ^13 ^C NMR (75 MHz, CDCl_3_): *δ* 68.8 (OCH_2_), 107.2 (Ar), 111.1 (Ar), 116.9 (C≡N), 123.1 (Ar), 124.7 (Ar), 127.2 (Ar), 128.5 (Ar), 129.0 (Ar), 132.7 (Ar), 133.0 (Ar), 133.5 (Ar), 138.4 (Ar), 138.8 (Ar), 139.3 (Ar), 150.4 (Ar), 158.9 (Ar). Anal. calcd for C_17_H_11_N_3_O_3_: C, 66.88; H, 3.63; N, 13.76, found: C, 66.40; H, 3.50; N, 13.61.

*5-nitro-8-((2-nitrobenzyl)oxy)quinoline* (**18**). Yellow solid, 82% yield, ClogP 3.61, mp = 208–210 °C. ^1^H NMR (300 MHz, CDCl_3_): *δ* 5.94 (*s*, 2H, OCH_2_), 7.07–7.10 (*m*, 1H, ArH), 7.55–7.57 (*m*, 1H, ArH), 7.72–7.77 (*m*, 2H, 2 × ArH), 8.02–8.04 (d, *J* = 7.5 Hz, 1H, ArH), 8.25 8.28 (d, *J* = 8.1 Hz, 1H, ArH), 8.46–8.49 (*m*, 1H, ArH), 9.10 (*s*, 1H, ArH), 9.23–9.26 (d, *J* = 9.0 Hz, 2H, 2 × ArH); ^13 ^C NMR (75 MHz, CDCl_3_): *δ* 68.3 (OCH_2_), 107.2 (Ar), 124.7 (Ar), 125.4 (Ar), 128.4 (Ar), 128.9 (Ar), 132.8 (Ar), 134.6 (Ar), 127.2 (Ar), 150.5 (Ar). Anal. calcd for C_16_H_11_N_3_O_5_: C, 59.08; H, 3.41; N, 12.92, found: C, 59.29; H, 3.57; N, 13.20.

*8-((2-bromobenzyl)oxy)-5-nitroquinoline* (**19**). Yellow solid, 74% yield, ClogP 4.81, mp = 144–146 °C. ^1^H NMR (300 MHz, CDCl_3_): *δ* 5.61 (*s*, 2H, OCH_2_), 7.00–7.03 (d, *J* = 8.7 Hz, 1H, ArH), 7.20–7.31 (*m*, 2H, 2 × ArH), 7.57–7.64 (*m*, 2H, 2 × ArH), 7.71–7.75 (*m*, H, ArH), 8.45–8.48 (d, *J* = 8.7 Hz, 1H, ArH), 9.10–9.11 (bs, 1H, ArH), 9.23–9.26 (d, *J* = 8.7 Hz, 1H, ArH); ^13 ^C NMR (75 MHz, CDCl_3_): *δ* 70.7 (OCH_2_), 107.3 (Ar), 122.0 (Ar), 123.1 (Ar), 124.7 (Ar), 127.4 (Ar), 127.9 (Ar), 128.6 (Ar), 129.8 (Ar), 132.7 (Ar), 132.9 (Ar), 134.5 (Ar), 138.0 (Ar), 139.5 (Ar), 150.4 (Ar), 159.3 (Ar). Anal. calcd for C_16_H_11_BrN_2_O_3_: C, 53.50; H, 3.09; N, 7.80, found: C, 53.12; H, 2.91; N, 7.55.

*8-((3-methylbenzyl)oxy)-5-nitroquinoline* (**20**). Yellow solid, 78% yield, ClogP 4.45, mp = 94–96 °C. ^1^H NMR (300 MHz, CDCl_3_): *δ* 2.35 (*s*, 3H, CH_3_), 5.51 (*s*, 2H, OCH_2_), 7.04–7.07 (d, *J* = 8.7 Hz, 1H, ArH), 7.14–7.15 (bs, 1H, ArH), 7.26–7.31 (ms, 3H, 3 × ArH), 7.68–7.73 (*m*, 1H, ArH), 8.42–8.45 (*m*, 1H, ArH), 9.07–9.08 (bs, 1H, ArH), 9.22–9.24 (*m*, 1H, ArH); ^13 ^C NMR (75 MHz, CDCl_3_): *δ* 21.4 (CH_3_), 71.6 (OCH_2_), 107.4 (Ar), 123.1 (Ar), 124.2 (Ar), 124.6 (Ar), 127.5 (Ar), 127.8 (Ar), 128.8 (Ar), 129.3 (Ar), 132.9 (Ar), 135.2 (Ar), 138.7 (Ar), 150.1 (Ar). Anal. calcd for C_17_H_14_N_2_O_3_: C, 69.38; H, 4.79; N, 9.52, found: C, 69.00; H, 4.52; N, 9.29.

*8-((3-methoxybenzyl)oxy)-5-nitroquinoline* (**21**). White solid, 85% yield, ClogP 3.87, mp = 100–101 °C. ^1^H NMR (400 MHz, CDCl_3_): *δ* 3.80 (bs, 3H, OCH_3_), 5.55 (*s*, 2H, OCH_2_), 6.88–6.90 (d, *J* = 8.0 Hz, 1H, ArH), 7.07–7.10 (*m*, 3H, 3 x ArH), 7.28–7.34 (*m*, 1H, ArH), 8.43–8.45 (*m*, 1H, ArH), 9.09 (bs, 1H, ArH), 9.22–9.24 (*m*, 1H, ArH); ^13 ^C NMR (100 MHz, CDCl_3_): *δ* 55.3 (OCH_3_), 71.4 (OCH_2_), 107.4 (Ar), 112.7 (Ar), 113.8 (Ar), 119.2 (Ar), 123.1 (Ar), 124.6 (Ar), 127.3 (Ar), 130.0 (Ar), 132.6 (Ar), 137.0 (Ar), 137.8 (Ar), 139.7 (Ar), 150.3 (Ar), 159.8 (Ar), 160.1 (Ar). Anal. calcd for C_17_H_14_N_2_O_4_: C, 65.80; H, 4.55; N, 9.03, found: C, 65.99; H, 4.60; N, 9.19.

*8-((3-fluorobenzyl)oxy)-5-nitroquinoline* (**22**). Yellow sticky oil, 65% yield, ClogP 4.09. ^1^H NMR (400 MHz, CDCl_3_): *δ* 5.55 (s, 2H, OCH_2_), 7.03–7.08 (*m*, 2H, 2 × ArH), 7.24–7.31 (*m*, 2H, 2 × ArH), 7.36–7.40 (*m*, 1H, ArH), 7.71–7.75 (*m*, 1H, ArH), 8.45–8.47 (d, *J* = 8.8 Hz, 1H, ArH), 9.09–9.11 (*m*, 1H, ArH), 9.23–9.25 (*m*, 1H, ArH); ^13 ^C NMR (100 MHz, CDCl_3_): *δ* 70.7 (OCH_2_), 107.2 (Ar), 114.1 (d, ^2^*J_C-F_* = 22.3 Hz, Ar), 115.5 (d, *^2^J_C-F_* = 21.2 Hz, Ar), 122.6 (d, ^4^*J_C-F_* = 3.1 Hz, Ar), 123.1 (Ar), 124.7 (Ar), 127.2 (Ar), 130.6 (d, ^3^*J_C-F_* = 8.3 Hz, Ar), 132.6 (Ar), 137.9 (Ar), 139.6 (Ar), 146.0 (Ar), 150.4 (Ar), 159.5 (Ar), 164.1 (Ar); ^19 ^F NMR (564.7 MHz, CDCl_3_): *δ* − 110.63 (td, F-H *J* = 9.0 Hz, 6.0 Hz, 1 F, ArF). Anal. calcd for C_16_H_11_FN_2_O_3_: C, 64.43; H, 3.72; N, 9.39, found: C, 64.69; H, 3.79; N, 9.48.

*8-((3-chlorobenzyl)oxy)-5-nitroquinoline* (**23**). Yellow solid, 82% yield, ClogP 4.66, mp = 126–128 °C. ^1^H NMR (300 MHz, CDCl_3_): *δ* 5.50 (*s*, 2H, OCH_2_), 7.01–7.04 (d, *J* = 8.7 Hz, 1H, ArH), 7.31–7.33 (*m*, 2H, 2 × ArH), 7.36–7.39 (*m*, 1H, ArH), 7.50 (*s*, 1H, ArH), 7.69–7.73 (*m*, 1H, ArH), 8.42–8.45 (d, *J* = 8.7 Hz, 1H, ArH), 9.06–9.08 (*m*, 1H, ArH), 9.20–9.23 (*m*, 1H, ArH); ^13 ^C NMR (75 MHz, CDCl_3_): *δ* 70.6 (OCH_2_), 107.2 (Ar), 123.1 (Ar), 124.7 (Ar), 125.2 (Ar), 127.2 (Ar), 128.7 (Ar), 130.3 (Ar), 132.7 (Ar), 134.9 (Ar), 137.3 (Ar), 139.5 (Ar), 150.4 (Ar), 159.4 (Ar). Anal. calcd for C_16_H_11_ClN_2_O_3_: C, 61.06; H, 3.52; N, 8.90, found: C, 61.48; H, 3.59; N, 8.99.

*8-((3-bromobenzyl)oxy)-5-nitroquinoline* (**24**). Beige solid, 87% yield, ClogP 4.81, mp = 141–143 °C. ^1^H NMR (300 MHz, CDCl_3_): *δ* 5.50 (*s*, 2H, OCH_2_), 7.04 (d, *J* = 8.7 Hz, 1H, ArH), 7.24–7.29 (*m*, 1H, ArH), 7.43–7.49 (*t*, *J* = 9.3 Hz, 2H, 2 × ArH), 7.66 (*s*, 1H, ArH), 7.70–7.74 (*m*, 1H, ArH), 8.44–8.47 (*m*, 1H, ArH), 9.08–9.09 (d, *J* = 3.0 Hz, 1H, ArH), 9.22–9.25 (d, *J* = 8.7 Hz, 1H, ArH); ^13 ^C NMR (75 MHz, CDCl_3_): *δ* 70.6 (OCH_2_), 107.2 (Ar), 123.0 (Ar), 123.1 (Ar), 124.7 (Ar), 125.7 (Ar), 127.2 (Ar), 130.1 (Ar), 130.5 (Ar), 131.6 (Ar), 132.7 (Ar), 137.6 (Ar), 138.0 (Ar), 139.5 (Ar), 150.4 (Ar), 151.3 (Ar), 159.4 (Ar). Anal. calcd for C_16_H_11_BrN_2_O_3_: C, 53.50; H, 3.09; N, 7.80, found: C, 53.19; H, 3.00; N, 7.67.

*5-nitro-8-((3-nitrobenzyl)oxy)quinoline* (**25**). Yellow sticky oil, 78% yield, ClogP 3.69. ^1^H NMR (300 MHz, CDCl_3_): *δ* 5.60 (*s*, 2H, OCH_2_), 7.06–7.09 (d, *J* = 8.7 Hz, 1H, ArH), 7.59–7.64 (*t*, *J* = 8.1 Hz, 1H, ArH), 7.72–7.76 (*m*, 1H, ArH), 7.88–7.91 (d, *J* = 7.5 Hz, 1H, ArH), 8.21–8.25 (*m*, 1H, ArH), 8.41 (*s*, 1H, ArH), 8.45–4.48 (d, *J* = 8.7 Hz, 1H, ArH), 9.08–9.10 (*m*, 1H, ArH), 9.21–9.25 (*m*, 1H, ArH); ^13 ^C NMR (75 MHz, CDCl_3_): *δ* 70.2 (OCH_2_), 107.2 (Ar), 122.3 (Ar), 123.6 (Ar), 124.8 (Ar), 127.1 (Ar), 130.1 (Ar), 132.8 (Ar), 133.2 (Ar), 137.4 (Ar), 150.5 (Ar). Anal. calcd for C_16_H_11_N_3_O_5_: C, 59.08; H, 3.41; N, 12.92, found: C, 59.49; H, 3.61; N, 13.07.

*5-nitro-8-((3-(trifluoromethyl)benzyl)oxy)quinoline* (**26**). Pink solid, 76% yield, ClogP 4.83, mp = 106–109 °C. ^1^H NMR (300 MHz, CDCl_3_): *δ* 5.56 (*s*, 2H, OCH_2_), 7.04–7.07 (d, *J* = 9.3 Hz, 1H, ArH), 7.51–7.56 (*m*, 1H, ArH), 7.60–7.62 (*m*, 1H, ArH), 7.70–7.78 (*m*, 3H, 3 × ArH), 8.44–8.47 (d, *J* = 9.0 Hz, 1H, ArH), 9.08–9.09 (*m*, 1H, ArH), 9.22–9.25 (*m*, 1H, ArH); ^13 ^C NMR (75 MHz, CDCl_3_): *δ* 70.8 (OCH_2_), 107.3 (Ar), 123.15 (Ar), 124.0 (d, ^4^*J*_C-F_ = 3.4 Hz, Ar), 124.7 (Ar), 125.4 (Ar), 125.5 (Ar), 127.2 (Ar), 129.5 (Ar), 130.6 (Ar), 132.9 (Ar), 136.3 (Ar), 150.3 (Ar), 159.2 (Ar); ^19 ^F NMR (564.7 MHz CDCl_3_): *δ* − 60.92 (s, 3 F, ArCF_3_). Anal. calcd for C_17_H_11_F_3_N_2_O_3_: C, 58.63; H, 3.18; N, 8.04, found: C, 58.96; H, 3.26; N, 8.15.

*8-((4-methylbenzyl)oxy)-5-nitroquinoline* (**27**). Yellow solid, 66% yield, ClogP 4.45, mp = 95–97 °C. ^1^H NMR (300 MHz, CDCl_3_): *δ* 2.20 (*s*, 3H, CH_3_), 5.36 (*s*, 2H, OCH_2_), 6.91–6.94 (d, *J* = 8.7 Hz, 1H, ArH), 7.05–7.07 (d, *J* = 7.5 Hz, 2H, 2 × ArH), 7.27–7.30 (d, *J* = 7.8 Hz, 2H, 2 × ArH), 7.49–7.52 (*m*, 1H, ArH), 8.23–8.26 (d, *J* = 9.0 Hz, 1H, ArH), 8.88 (*s*, 1H, ArH), 8.99–9.02 (d, *J* = 9.0 Hz, 1H, ArH); ^13 ^C NMR (75 MHz, CDCl_3_): *δ* 21.1 (CH_3_), 71.3 (OCH_2_), 107.2 (Ar), 122.8 (Ar), 124.4 (Ar), 127.3 (Ar), 129.4 (Ar), 132.2 (Ar), 137.3 (Ar), 138.1 (Ar), 139.3 (Ar), 150.0 (Ar), 159.7 (Ar). Anal. calcd for C_17_H_14_N_2_O_3_: C, 69.38; H, 4.79; N, 9.52, found: C, 69.59; H, 4.87; N, 9.69.

*8-((4-(methylthio)benzyl)oxy)-5-nitroquinoline* (**28**). Brown solid, 71% yield, ClogP 4.51, mp = 123–128 °C. ^1^H NMR (300 MHz, CDCl_3_): *δ* 2.43 (*s*, 3H, SCH_3_), 5.46 (*s*, 2H, OCH_2_), 7.02–7.05 (d, *J* = 9.0 Hz, 1H, ArH), 7.20–7.23 (d, *J* = 8.4 Hz, 2H, 2 × ArH), 7.38–7.41 (d, *J* = 8.1 Hz, 2H, 2 × ArH), 7.66–7.70 (*m*, 1H, ArH), 8.39–8.42 (d, *J* = 8.7 Hz, 1H, ArH), 9.03 (*s*, 1H, ArH), 9.18–9.21 (d, *J* = 8.1 Hz, 1H, ArH); ^13 ^C NMR (75 MHz, CDCl_3_): *δ* 22.0 (SCH_3_), 77.6 (OCH_2_), 114.0 (Ar), 129.6 (Ar), 131.0 (Ar), 133.0 (Ar), 134.1 (Ar), 134.5 (Ar), 138.2 (Ar), 139.0 (Ar), 140.2 (Ar), 144.1 (Ar), 145.8 (Ar), 155.8 (Ar), 156.3 (Ar), 157.8 (Ar), 166.0 (Ar). Anal. calcd for C_17_H_14_N_2_O_3_S: C, 62.56; H, 4.32; N, 8.58, found: C, 62.82; H, 4.41; N, 8.69.

*8-((4-fluorobenzyl)oxy)-5-nitroquinoline* (**29**). Yellow solid, 69% yield, ClogP 4.09, mp = 177–179 °C. ^1^H NMR (300 MHz, CDCl_3_): *δ* 5.48 (*s*, 2H, OCH_2_), 7.04–7.11 (*m*, 3H, ArH), 7.47–7.52 (*t*, *J* = 6.9 Hz, 2H, ArH), 7.68–7.72 (*m*, 1H, ArH), 8.43–8.46 (d, *J* = 8.7 Hz, 1H, ArH), 9.05–9.06 (*s*, *J* = 2.1 Hz, 1H, ArH), 9.20–9.22 (d, *J* = 9.0 Hz, 1H, ArH); ^13 ^C NMR (75 MHz, CDCl_3_): *δ* 70.9 (OCH_2_), 107.5 (Ar), 115.9 (d, ^2^*J*_C-F_ = 21.75 Hz, 2 × Ar), 123.1 (Ar), 124.7 (Ar), 127.5 (Ar), 129.3 (d, ^3^*J*_C-F_ = 7.95 Hz, 2 x Ar), 130.9 (Ar), 133.4 (Ar), 137.8 (Ar), 150.0 (Ar), 159.2 (Ar), 161.1 (Ar-F), 164.4 (Ar); ^19 ^F NMR (564.7 MHz, CDCl_3_): *δ* −111.83 (*m*, 1 F, ArF). Anal. calcd for C_16_H_11_FN_2_O_3_: C, 64.43; H, 3.72; N, 9.39, found: C, 64.02; H, 3.62; N, 9.25.

*8-((4-chlorobenzyl)oxy)-5-nitroquinoline* (**30**). Yellow solid, 81% yield, ClogP 4.66, mp = 152–153 °C. ^1^H NMR (300 MHz, DMSO-*d*_6_): *δ* 5.46 (*s*, 2H, OCH_2_), 7.42–7.60 (*m*, 5H, 5 × ArH), 7.81–7.86 (*m*, 1H, ArH), 8.54–8.57 (d, *J* = 8.7 Hz, 1H, ArH), 9.00–9.03 (*m*, 2H, 2 × ArH); ^13 ^C NMR (75 MHz, DMSO-*d*_6_): *δ* 70.3 (OCH_2_), 108.3 (Ar), 125.3 (Ar), 128.1 (Ar), 129.1 (Ar), 130.3 (Ar), 132.2 (Ar), 135.5 (Ar), 150.8 (Ar). Anal. calcd for C_16_H_11_ClN_2_O_3_: C, 61.06; H, 3.52; N, 8.90, found: C, 61.38; H, 3.65; N, 9.12.

*8-((4-bromobenzyl)oxy)-5-nitroquinoline* (**31**). Beige solid, 85% yield, ClogP 4.81, mp = 165–167 °C. ^1^H NMR (300 MHz, CDCl_3_): *δ* 5.48 (*s*, 2H, OCH_2_), 7.00–7.04 (*m*, 1H, ArH), 7.37–7.40 (*m*, 2H, 2 × ArH), 7.51–7.54 (*m*, 2H, 2 × ArH), 7.69–7.74 (*m*, 1H, ArH), 8.42–8.46 (*m*, 1H, ArH), 9.07–9.08 (*m*, 1H, ArH), 9.21–9.25 (*m*, 1H, ArH); ^13 ^C NMR (75 MHz, CDCl_3_): *δ* 70.7 (OCH_2_), 107.2 (Ar), 122.4 (Ar), 123.1 (Ar), 124.6 (Ar), 127.2 (Ar), 128.9 (Ar), 132.1 (Ar), 132.6 (Ar), 134.3 (Ar), 137.9 (Ar), 139.5 (Ar), 150.3 (Ar), 159.4 (Ar). Anal. calcd for C_16_H_11_BrN_2_O_3_: C, 53.50; H, 3.09; N, 7.80, found: C, 53.19; H, 3.00; N, 7.69.

*8-((4-iodobenzyl)oxy)-5-nitroquinoline* (**32**). Brown solid, 78% yield, ClogP 5.07, mp = 195–197 °C. ^1^H NMR (400 MHz, DMSO-*d*_6_): *δ* 5.44 (*s*, 2H, OCH_2_), 7.37–7.44 (*m*, 3H, ArH), 7.81–7.86 (*m*, 3H, ArH), 8.55–8.57 (d, *J* = 8.8 Hz, 1H, ArH), 9.02–9.04 (d, *J* = 8.8 Hz, 2H, ArH); ^13 ^C NMR (100 MHz, DMSO-*d*_6_): *δ* 70.5 (OCH_2_), 108.2 (Ar), 122.6 (Ar), 125.3 (Ar), 128.1 (Ar), 129.2 (Ar), 130.6 (Ar), 132.2 (Ar), 136.2 (Ar), 137.2 (Ar), 137.7 (Ar), 137.8 (Ar), 139.2 (Ar), 150.8 (Ar), 159.8 (Ar). Anal. calcd for C_16_H_11_IN_2_O_3_: C, 47.31; H, 2.73; N, 6.90, found: C, 47.58; H, 2.80; N, 6.99.

*4-(((5-nitroquinolin-8-yl)oxy)methyl)benzonitrile* (**33**). Brown solid, 94% yield, ClogP 3.38, mp = 208–212 °C. ^1^H NMR (300 MHz, DMSO-*d*_6_): *δ* 5.57 (*s*, 2H, OCH_2_), 7.39–7.43 (*m*, 1H, ArH), 7.73–7.76 (d, *J* = 8.4 Hz, 2H, 2 × ArH), 7.81–7.86 (*m*, 1H, ArH), 7.90–7.93 (*m*, 2H, 2 × ArH), 8.52–8.55 (*m*, 1H, ArH), 8.99–9.03 (*m*, 2H, 2 × ArH); ^13 ^C NMR (75 MHz, DMSO-*d*_6_): *δ* 70.1 (OCH_2_), 108.3 (Ar), 111.3 (Ar), 119.2 (CN), 122.6 (Ar), 125.3 (Ar), 128.0 (Ar), 128.7 (Ar), 132.2 (Ar), 133.0 (Ar), 138.0 (Ar), 139.2 (Ar), 142.2 (Ar), 150.9 (Ar), 159.6 (Ar). Anal. calcd for C_17_H_11_N_3_O_3_: C, 66.88; H, 3.63; N, 13.76, found: C, 67.00; H, 3.70; N, 13.82.

*5-nitro-8-((4-(trifluoromethyl)benzyl)oxy)quinoline* (**34**). Brown solid, 81% yield, ClogP 4.83, mp = 128–130 °C. ^1^H NMR (300 MHz, CDCl_3_): *δ* 5.57 (*s*, 2H, CH_2_), 6.99–7.02 (d, *J* = 8.7 Hz, 1H, ArH), 7.64–7.71 (*m*, 5H, 5 × ArH), 8.40–8.43 (d, *J* = 9.0 Hz, 1H, ArH), 9.05–9.06 (*m*, 1H, ArH), 9.17–9.20 (*m,* 1H, ArH); ^13 ^C NMR (75 MHz, CDCl_3_): *δ* 70.6 (OCH_2_), 107.3 (Ar), 123.1 (Ar), 123.9 (d, ^1^*J_C-F_* = 270.0 Hz, CF_3_), 124.7 (Ar), 125.9 (q, ^3^*J_C-F_* = 4.01 Hz, 2 × Ar), 126.6 (Ar), 127.2 (d, ^4^*J_C-F_* = 4.6 Hz, 2 x Ar), 130.6 (q, ^2^*J_C-F_* = 31.2 Hz, Ar), 132.9 (Ar), 138.1 (Ar), 139.2 (Ar), 139.3 (Ar), 150.3 (Ar), 159.1 (Ar). ^19 ^F NMR (564.7 MHz, CDCl_3_): *δ* − 61.32 (s, 3 F, ArCF_3_). Anal. calcd for C_17_H_11_F_3_N_2_O_3_: C, 58.63; H, 3.18; N, 8.04, found: C, 58.49; H, 3.15; N, 7.96.

*5-nitro-8-((4-nitrobenzyl)oxy)quinoline* (**35**). Yellow sticky oil, 98% yield, ClogP 3.69. ^1^H NMR (300 MHz, CDCl_3_): *δ* 5.52 (*s*, 2H, OCH_2_), 7.35–7.38 (*m*, 5H, 5 × ArH), 8.05–8.08 (*m*, 4H, 4 × ArH); ^13 ^C NMR (75 MHz, CDCl_3_): *δ* 70.1 (OCH_2_), 108.4 (Ar), 111.4 (Ar), 122.5 (Ar), 125.4 (Ar), 128.7 (Ar), 133.1 (Ar), 138.2 (Ar), 139.0 (Ar), 142.4 (Ar), 150.7 (Ar), 159.9 (Ar). Anal. calcd for C_16_H_11_N_3_O_5_: C, 59.08; H, 3.41; N, 12.92, found: C, 59.28; H, 3.49; N, 12.99.

*8-((2,6-difluorobenzyl)oxy)-5-nitroquinoline* (**36**). Yellow solid, 88% yield, ClogP 4.24, mp = 193–195 °C. ^1^H NMR (300 MHz, CDCl_3_): *δ* 5.52 (*s*, 2H, OCH_2_), 6.94–6.99 (*t*, *J* = 7.8 Hz, 2H, 2 × ArH), 7.26–7.29 (d, *J* = 9.3 Hz, 1H, ArH), 7.32–7.40 (*m*, 1H, ArH), 7.65–7.69 (*m*, 1H, ArH), 8.52–8.55 (d, *J* = 8.7 Hz, 1H, ArH), 9.03–9.05 (d, *J* = 4.2 Hz, 1H, ArH), 9.20 (d, *J* = 9.0 Hz, 1H, ArH); ^13 ^C NMR (75 MHz, CDCl_3_) *δ* 59.7 (OCH_2_), 106.6 (Ar), 111.6 (Ar), 111.8 (d, ^2^*J*_C-F_ = 22.9 Hz, 2 x Ar), 123.1 (Ar), 124.5 (Ar), 127.3 (Ar), 131.4 (Ar), 131.6 (d, ^3^*J*_C-F_ = 10.3 Hz, Ar), 132.6 (Ar), 139.8 (Ar), 143.4 (Ar), 150.3 (Ar), 159.7 (Ar), 160.4 (ArC-F x 2). ^19 ^F NMR (564.7 MHz, CDCl_3_): *δ* − 111.74 (t, *J* = 6.6 Hz, 2 F, ArF). Anal. calcd for C_16_H_10_F_2_N_2_O_3_: C, 60.76; H, 3.19; N, 8.86, found: C, 60.89; H, 3.22; N, 8.95.

*8-((3,5-dimethylbenzyl)oxy)-5-nitroquinoline* (**37**). Brownish sticky oil, 81% yield, ClogP 4.95. ^1^H NMR (400 MHz, CDCl_3_): *δ* 2.32 (*s*, 6H, 2 x CH_3_), 5.49 (*s*, 2H, OCH_2_), 6.98 (*s*, 1H, ArH), 7.07–7.09 (d, *J* = 8.8 Hz, 1H, ArH), 7.12 (*s*, 1H, ArH), 7.69–7.73 (*m*, 1H, ArH), 8.44–8.46 (d, *J* = 8.8 Hz, 1H, ArH), 9.08–9.09 (*m*, 1H, ArH), 9.22–9.25 (*m* 1H, ArH); ^13 ^C NMR (100 MHz, CDCl_3_): *δ* 21.3 (2 × CH_3_), 71.7 (OCH_2_), 107.3 (Ar), 123.1 (Ar), 124.5 (Ar), 124.9 (Ar), 127.5 (Ar), 130.1 (Ar), 132.6 (Ar), 135.2 (Ar), 137.7 (Ar), 138.6 (Ar), 139.6 (Ar), 150.3 (Ar), 160.0 (Ar). Anal. calcd for C_18_H_16_N_2_O_3_: C, 70.12; H, 5.23; N, 9.09, found: C, 70.29; H, 5.27; N, 9.18.

*8-((3,5-difluorobenzyl)oxy)-5-nitroquinoline* (**38**). Yellow solid, 71% yield, ClogP 4.24, mp = 135–136 °C. ^1^H NMR (400 MHz, CDCl_3_): *δ* 5.51 (*s*, 2H, OCH_2_), 6.78–6.83 (*m*, 1H, ArH), 7.02–7.09 (*m*, 3H, 3 × ArH), 7.72–7.75 (*m*, 1H, ArH), 8.45–8.47 (d, *J* = 8.8 Hz, 1H, ArH), 9.09–9.11 (*m*, 1H, ArH), 9.22–9.24 (dd, *J* = 1.5 Hz, *J* = 8.8 Hz, 1H, ArH); ^13 ^C NMR (100 MHz, CDCl_3_): *δ* 70.1 (OCH_2_), 103.9 (t, *^2^J_C-F_* = 25.3 Hz, Ar), 107.1 (Ar), 109.8 (d, *^2^J_C-F_* = 26.1 Hz, 2 × Ar), 123.1 (Ar), 124.7 (Ar), 127.0 (Ar), 132.6 (Ar), 138.3 (Ar), 139.4 (t, *^3^J_C-F_* = 9.1 Hz, Ar), 139.6 (Ar), 150.5 (Ar), 159.1 (Ar), 163.3 (d, *^1^J_C-F_* = 249.7 Hz, ArC-F), 163.4 (d, *^1^J_C-F_* = 249.8 Hz, ArC-F). Anal. calcd for C_16_H_10_F_2_N_2_O_3_: C, 60.76; H, 3.19; N, 8.86, found: C, 60.50; H, 3.13; N, 8.77.

*8-((3-chloro-5-methoxybenzyl)oxy)-5-nitroquinoline* (**39**). Yellow solid, 72% yield, ClogP 4.72, mp = 154–155 °C. ^1^H NMR (400 MHz, DMSO-*d*_6_): *δ* 3.82 (*s*, 3H, OCH_3_), 5.42 (*s*, 2H, OCH_2_), 7.01–7.03 (d, *J* = 8.8 Hz, 1H, ArH), 7.15–7.16 (d, *J* = 2.8 Hz, 1H, ArH), 7.50–7.52 (d, *J* = 8.8 Hz, 1H, ArH), 7.65–7.67 (d, *J* = 8.4 Hz, 1H, ArH), 7.82–7.85 (*m*, 1H, ArH), 8.56–8.59 (d, *J* = 9.2 Hz, 1H, ArH), 8.98–9.04 (*m* 2H, 2 × ArH); ^13 ^C NMR (100 MHz, DMSO-*d*_6_): *δ* 56.2 (OCH_3_), 68.8 (OCH_2_), 108.0 (Ar), 113.9 (Ar), 115.3 (Ar), 122.6 (Ar), 125.3 (Ar), 125.6 (Ar), 128.2 (Ar), 132.2 (Ar), 132.6 (Ar), 134.7 (Ar), 137.8 (Ar), 139.2 (Ar), 150.7 (Ar), 160.0 (Ar), 160.8 (Ar). Anal. calcd for C_17_H_13_ClN_2_O_4_: C, 59.23; H, 3.80; N, 8.13, found: C, 59.39; H, 3.86; N, 8.20.

*8-((3,4-dichlorobenzyl)oxy)-5-nitroquinoline* (**40**). Red solid, 78% yield, ClogP 5.26, mp = 122–132 °C. ^1^H NMR (300 MHz, CDCl_3_): *δ* 5.44 (*s*, 2H, OCH_2_), 6.99–7.02 (d, *J* = 9.0 Hz, 1H, ArH), 7.32–7.34 (d, *J* = 8.4 Hz, 1H, ArH), 7.43–7.46 (d, *J* = 8.1 Hz, 1H, ArH), 7.59 (*s*, 1H, ArH), 7.67–7.71 (*m*, 1H, ArH), 8.40–8.43 (d, *J* = 9.0 Hz, 1H, ArH), 9.03–9.04 (*m*, 1H, ArH), 9.16–9.19 (d, *J* = 8.7 Hz, 1H; ArH); ^13 ^C NMR (75 MHz, CDCl_3_): *δ* 70.0 (OCH_2_), 107.1 (Ar), 123.1 (Ar), 124.7 (Ar), 126.5 (Ar), 127.2 (Ar), 129.1 (Ar), 130.9 (Ar), 132.6 (Ar), 133.1 (Ar), 135.5 (Ar), 138.1 (Ar), 139.4 (Ar), 150.5 (Ar), 159.1 (Ar). Anal. calcd for C_16_H_10_Cl_2_N_2_O_3_: C, 55.04; H, 2.89; N, 8.02, found: C, 55.29; H, 2.98; N, 8.11.

*8-(naphthalen-1-ylmethoxy)-5-nitroquinoline* (**41**). Red solid, 81% yield, ClogP 5.12, mp = 177.0–178.0 °C. ^1^H NMR (300 MHz, CDCl_3_): *δ* 5.87 (*s*, 2H, OCH_2_), 7.52–7.57 (*m*, 3H, 3 × ArH), 7.61–7.64 (d, *J* = 9.0 Hz, 1H, ArH), 7.74–7.81 (*m*, 2H, 2 × ArH), 7.95–7.99 (*m*, 2H, 2 × ArH), 8.11–8.14 (*m*, 1H, ArH), 8.55–8.58 (d, *J* = 8.7 Hz, 1H, ArH), 8.89–8.90 (d, *J* = 3.9 Hz, 1H, ArH), 8.97–9.00 (*m*, 1H, ArH); ^13 ^C NMR (75 MHz, CDCl_3_): *δ* 69.8 (OCH_2_), 108.3 (Ar), 122.6 (Ar), 124.4 (Ar), 125.3 (Ar), 125.9 (Ar), 126.6 (Ar), 127.0 (Ar), 127.6 (Ar), 128.2 (Ar), 129.0 (Ar), 129.5 (Ar), 131.7 (Ar), 131.9 (Ar), 132.1 (Ar), 133.8 (Ar), 137.7 (Ar), 139.2 (Ar), 150.6 (Ar), 160.1 (Ar). Anal. calcd for C_20_H_14_N_2_O_3_: C, 72.72; H, 4.27; N, 8.48, found: C, 72.89; H, 4.31; N, 8.59.

*2-(((5-nitroquinolin-8-yl)oxy)methyl)isoindoline-1,3-dione* (**42**). Yellow solid, 77% yield, ClogP 3.07, mp = 210 °C (dec.); ^1^H NMR (400 MHz, CDCl_3_): *δ* 5.92 (*s*, 2H, OCH_2_), 7.64–7.67 (d, *J* = 8.7 Hz, 1H, ArH), 7.80–7.84 (*m*, 1H, ArH), 7.91–8.00 (*m*, 4H, 4 × ArH), 8.53–8.56 (d, *J* = 8.7 Hz, 1H, ArH), 8.94–8.99 (*m*, 2H, 2 × ArH); ^13 ^C NMR (100 MHz, CDCl_3_): *δ* 67.0 (OCH_2_), 110.5 (Ar), 122.5 (Ar), 124.3 (Ar), 127.5 (Ar), 131.7 (Ar), 132.3 (Ar), 135.7 (Ar), 139.1 (Ar), 151.0 (Ar), 157.8 (Ar), 167.3 (2 x N-C = O). Anal. calcd for C_18_H_11_N_3_O_5_: C, 61.89; H, 3.17; N, 12.03, found: C, 61.70; H, 3.13; N, 11.96.

*2-((5-nitroquinolin-8-yl)oxy)-1-phenylethan-1-one* (**43**). Yellow-brow solid, 81% yield, ClogP 3.14, mp = 196–198 °C. ^1^H NMR (300 MHz, CDCl_3_): *δ* 5.80 (*s*, 2H, OCH_2_), 6.90–6.92 (d, *J* = 9.0 Hz, 1H, ArH), 7.51–7.56 (*t*, *J* = 7.8 Hz, 2H, 2 × ArH), 7.63–7.66 (d, *J* = 6.9 Hz, 1H, ArH), 7.69–7.74 (*m*, 1H, ArH), 8.03–8.06 (d, *J* = 7.8 Hz, 2H, 2 × ArH), 8.42–8.45 (d, *J* = 8.7 Hz, 1H, ArH), 9.06–9.08 (d, *J* = 3.6 Hz, 1H, ArH), 9.22–9.25 (d, *J* = 8.7 Hz, 1H, ArH); ^13 ^C NMR (75 MHz, DMSO-*d*_6_): *δ* 71.8 (OCH_2_), 108.5 (Ar), 122.6 (Ar), 123.3 (Ar), 125.3 (Ar), 127.9 (Ar), 128.5 (Ar), 129.4 (Ar), 132.2 (Ar), 134.5 (Ar), 137.8 (Ar), 138.9 (Ar), 150.7 (Ar), 159.8 (Ar), 193.7 (C = O). Anal. calcd for C_17_H_12_N_2_O_4_: C, 66.23; H, 3.92; N, 9.09, found: C, 66.36; H, 3.99; N, 9.19.

*1–(3-nitrophenyl)-2-((5-nitroquinolin-8-yl)oxy)ethan-1-one* (**44**). Dark brown solid, 65% yield, ClogP 3.05, mp = 203–205 °C. ^1^H NMR (300 MHz, DMSO-*d*_6_, slightly soluble): *δ* 5.82 (*s*, 2H, OCH_2_), 7.07–7.10 (d, *J* = 8.4 Hz, 1H, ArH), 7.76–7.85 (*m*, 2H, 2 × ArH), 8.48–8.53 (*m*, 3H, 3 x ArH), 8.92 (*s*, 1H, ArH), 9.15 (*s*, 1H, ArH), 9.37–9.39 (*m*, 1H, ArH). Anal. calcd for C_17_H_11_N_3_O_6_: C, 57.80; H, 3.14; N, 11.89, found: C, 57.96; H, 3.18; N, 11.99.

*1–(4-nitrophenyl)-2-((5-nitroquinolin-8-yl)oxy)ethan-1-one* (**45**). Brown solid, 73% yield, ClogP 3.05, mp = 190 °C (dec.). ^1^H NMR (300 MHz, DMSO-*d*_6_, slightly soluble): *δ* 6.11 (*s*, 2H, OCH_2_), 7.38–7.41 (d, *J* = 8.7 Hz, 1H, ArH), 7.83–7.87 (*m*, 1H, ArH), 8.27–8.30 (d, *J* = 9.0 Hz, 2H, 2 × ArH), 8.39–8.42 (d, *J* = 8.7 Hz, 2H, 2 × ArH), 8.46–8.49 (d, *J* = 9.3 Hz, 1H, ArH), 9.01–9.03 (*m*, 2H, 2 × ArH). Anal. calcd for C_17_H_11_N_3_O_6_: C, 57.80; H, 3.14; N, 11.89, found: C, 57.62; H, 3.10; N, 11.78.

*2-((5-nitroquinolin-8-yl)oxy)-1-(thiophen-3-yl)ethan-1-one* (**46**). Brown solid, 86% yield, ClogP 2.92, mp = 128–130 °C. ^1^H NMR (400 MHz, DMSO-*d*_6_): *δ* 5.89 (*s*, 2H, OCH_2_), 7.29–7.31 (d, *J* = 9.2 Hz, 1H, ArH), 7.63–7.64 (d, *J* = 5.2 Hz, 1H, ArH), 7.72–74 (*m*, 1H, ArH), 7.85–7.89 (*m*, 1H, ArH), 8.50–8.52 (d, *J* = 8.8 Hz, 1H, ArH), 8.79–8.80 (d, *J* = 1.6 Hz, 1H, ArH), 9.03–7.06 (*m*, 2H, 2 x ArH); ^13 ^C NMR (100 MHz, DMSO*-d*_6_): *δ* 72.0 (OCH_2_), 108.4 (Ar), 122.6 (Ar), 125.3 (Ar), 127.0 (thiophene), 127.9 (thiophene), 128.2 (Ar), 132.2 (thiophene), 135.0 (Ar), 137.9 (Ar), 139.0 (Ar), 139.1 (thiophene), 150.7 (Ar), 159.7 (Ar), 188.4 (C = O). Anal. calcd for C_15_H_10_N_2_O_4_S: C, 57.32; H, 3.21; N, 8.91, found: C, 57.50; H, 3.26; N, 8.99.

*1-bromo-2-((4-nitrophenoxy)methyl)benzene* (**47**). White solid, 89% yield, ClogP 4.73, mp = 122–123 °C. Characterisation data are in agreement with those reported in the literature^25^.

*1-nitro-2-((4-nitrophenoxy)methyl)benzene* (**48**). Pinkish solid, 97% yield, ClogP 3.53, mp = 130–132 °C. ^1^H NMR (400 MHz, CDCl_3_): *δ* 5.62 (*s*, 2H, OCH_2_), 7.08–7.12 (*m*, 2H, 2 × ArH), 7.55–7.59 (t, *J* = 8.0 Hz, 1H, ArH), 7.73–7.77 (*t*, *J* = 7.6 Hz, 1H, ArH), 7.85–7.87 (d, *J* = 8.0 Hz, 1H, ArH), 8.22–8.27 (*t*, *J* = 9.2 Hz, 3H, 3 × ArH); ^13 ^C NMR (100 MHz, CDCl_3_): *δ* 67.4 (OCH_2_), 115.0 (Ar), 125.3 (Ar), 126.1 (Ar), 129.0 (Ar), 132.2 (Ar), 134.2 (Ar), 142:2 (Ar), 147.0 (Ar), 163.0 (Ar). Anal. calcd for C_13_H_10_N_2_O_5_: C, 56.94; H, 3.68; N, 10.22, found: C, 56.74; H, 3.61; N, 10.14.

*1-bromo-3-((4-nitrophenoxy)methyl)benzene* (**49**). White solid, 90% yield, ClogP 4.73, mp = 91–92 °C. Characterisation data are in agreement with those reported in the literature^26^.

*1-nitro-3-((4-nitrophenoxy)methyl)benzene* (**50**). Yellow solid, 60% yield, ClogP 3.61, mp = 125–128 °C. ^1^H NMR (400 MHz, CDCl_3_): *δ* 5.28 (*s*, 2H, OCH_2_), 7.07–7.11 (*m*, 2H, 2 × ArH), 7.64–7.66 (*t*, *J* = 8.0 Hz, 1H, ArH), 7.79–7.81 (d, *J* = 7.6 Hz, 1H, ArH), 8.25–8.27 (d, *J* = 9.2 Hz, 3H, 3 × ArH), 8.35 (*s*, 1H, ArH); ^13 ^C NMR (100 MHz, CDCl_3_): *δ* 69.2 (OCH_2_), 114.9 (Ar), 122.3 (Ar), 123.5 (Ar), 126.1 (Ar), 129.9 (Ar), 133.1 (Ar), 137.7 (Ar), 142.2 (Ar), 148.6 (Ar), 163.0 (Ar). Anal. calcd for C_13_H_10_N_2_O_5_: C, 56.94; H, 3.68; N, 10.22, found: C, 56.74; H, 3.60; N, 10.16.

*1-((4-nitrophenoxy)methyl)-3-(trifluoromethyl)benzene* (**51**). White solid, 72% yield, ClogP 4.75, mp = 70–74 °C. ^1^H NMR (400 MHz, CDCl_3_): *δ* 5.23 (*s*, 2H, OCH_2_), 7.05–7.09 (*m*, 2H, 2 x ArH), 7.57–7.73 (*m*, 4H, 4 x ArH), 8.23–8.27 (*m*, 2H, 2 x ArH);^19^F NMR (564.7 MHz, CDCl_3_): *δ* − 66.64 (*s*, 3 F, ArCF_3_) Anal. calcd for C_14_H_10_F_3_NO_3_: C, 56.57; H, 3.39; N, 4.71, found: C, 56.64; H, 3.41; N, 4.73.

*(3-nitrobenzyl)(4-nitrophenyl)sulfane* (**52**). Yellow sticky oil, 70% yield, ClogP 3.87. ^1^H NMR (400 MHz, CDCl_3_): *δ* 4.26 (*s*, 2H, SCH_2_), 7.26–7.30 (*m*, 2H, 2 × ArH), 7.43–7.47 (*t*, *J* = 8.0 Hz, 1H, ArH), 7.63–7.65 (dd, *J* = 7.6 Hz, *J* = 0.4 Hz, 1H, ArH), 8.02–8.08 (*m*, 1H, ArH), 8.19–8.20 (*t*, *J* = 1.6 Hz, 3H, 3 × ArH); ^13 ^C NMR (100 MHz, CDCl_3_): *δ* 36.5 (SCH_2_), 122.9 (Ar), 123.6 (Ar), 124.1 (Ar), 127.3 (Ar), 129.9 (Ar), 134.7 (Ar), 138.0 (Ar), 145.5 (Ar), 145.7 (Ar), 148.5 (Ar). Anal. calcd for C_13_H_10_N_2_O_4_S: C, 53.79; H, 3.47; N, 9.65, found: C, 53.27; H, 3.43; N, 9.60.

*1-fluoro-4-((4-nitrophenoxy)methyl)benzene* (**53**). White solid, 92% yield, ClogP 4.01, mp = 123–124 °C. [Lit. mp = 122.4 °C]. ^19 ^F NMR (564.7 MHz, CDCl_3_): *δ* − 110.05 (*m*, 1 F, ArF). Characterisation data are in agreement with those reported in the literature^27^.

*1-chloro-4-((4-nitrophenoxy)methyl)benzene* (**54**). White solid, 98% yield, ClogP 4.58, mp = 113–115 °C. [Lit. mp = 114–115 °C]. Characterisation data are in agreement with those reported in the literature^28^.

*1-bromo-4-((4-nitrophenoxy)methyl)benzene* (**55**). White solid, 89% yield, ClogP 4.73, mp = 120–121 °C. ^1^H NMR (400 MHz, CDCl_3_): *δ* 5.13 (*s*, 2H, OCH_2_), 7.03–7.05 (*m*, 2H, 2 × ArH), 7.32–7.34 (d, *J* = 8.4 Hz, 2H, 2 x ArH), 7.56–7.58 (d, *J* = 8.4 Hz, 2H, 2 × ArH), 8.22–8.25 (*m*, 2H, 2 × ArH). Anal. calcd for C_13_H_10_BrNO_3_: C, 50.67; H, 3.27; N, 4.55, found: C, 50.69; H, 3.29; N, 4.58.

*4-((4-nitrophenoxy)methyl)benzonitrile* (**56**). White solid, 94% yield, ClogP 3.30, mp = 157–158 °C. ^1^H NMR (400 MHz, CDCl_3_): *δ* 5.25 (*s*, 2H, OCH_2_), 7.03–7.07 (*m*, 2H, 2 × ArH), 7.57–7.59 (d, *J* = 8.4 Hz, 2H, 2 × ArH), 7.72–7.74 (d, *J* = 8.4 Hz, 2H, 2 × ArH), 8.22–8.26 (*m*, 2H, 2 × ArH); ^13 ^C NMR (100 MHz, CDCl_3_): *δ* 69.5 (OCH_2_), 112.4 (Ar), 114.8 (Ar), 118.4 (C≡N), 126.0 (Ar), 127.7 (Ar), 132.6 (Ar), 140.9 (Ar), 142.1 (Ar), 163.0 (Ar). Anal. calcd for C_14_H_10_N_2_O_3_: C, 66.14; H, 3.96; N, 11.02, found: C, 66.26; H, 3.99; N, 11.11.

*(4-bromobenzyl)(4-nitrophenyl)sulfane* (**57**). Yellow solid, 87% yield, ClogP 4.99, mp = 133–134° C. ^1^H NMR (400 MHz, CDCl_3_): *δ* 4.21 (*s*, 2H, SCH_2_), 7.27–7.29 (d, *J* = 8.4 Hz, 2H, 2 × ArH), 7.33–7.7.35 (*m*, 2H, 2 × ArH), 7.47–7.49 (*m*, 2H, 2 × ArH), 8.11–8.13 (*m*, 2H, 2 × ArH); ^13 ^C NMR (100 MHz, CDCl_3_): *δ* 36.5 (SCH_2_), 121.8 (Ar), 124.0 (Ar), 126.9 (Ar), 130.4 (Ar), 132.0 (Ar), 134.6 (Ar), 145.5 (Ar), 146.5 (Ar). Anal. calcd for C_13_H_10_BrNO_2_S: C, 48.16; H, 3.11; N, 4.32, found: C, 48.29; H, 3.14; N, 4.39.

*1,3-difluoro-2-((4-nitrophenoxy)methyl)benzene* (**58**). White solid, 95% yield, ClogP 4.15, mp = 125–127 °C. ^1^H NMR (400 MHz, DMSO-*d*_6_): *δ* 5.29 (*s*, 2H, OCH_2_), 7.18–7.23 (*m*, 2H, 2 × ArH), 7.26–7.29 (d, *J* = 9.2 Hz, 2H, 2 × ArH), 7.53–7.61 (*m*, 1H, ArH), 8.23–8.25 (d, *J* = 9.2 Hz, 2H, 2 × ArH); ^13 ^C NMR (100 MHz, DMSO-*d*_6_): *δ* 59.0 (OCH_2_), 111.9 (t, ^2^*J*_C-F_ = 19.1 Hz, Ar), 112.4 (d, ^2^*J*_C-F_ = 24.8 Hz, Ar), 115.7 (2 x Ar), 126.4 (2 x Ar), 132.6 (*t*, ^3^*J*_C-F_ = 10.5 Hz, Ar), 141.8 (Ar), 161.6 (d, ^1^*J*_C-F_ = 249.6 Hz, ArC-F), 161.7 (d, ^1^*J*_C-F_ = 249.6 Hz, ArC-F), 163.7 (Ar); ^19 ^F NMR (564.7 MHz, CDCl_3_): *δ* − 118.37 (t, *J* = 6.6 Hz, 2 F, ArF). Anal. calcd for C_13_H_9_F_2_NO_3_: C, 58.87; H, 3.42; N, 5.28, found: C, 58.99; H, 3.46; N, 5.32.

*1,2-dichloro-4-((4-nitrophenoxy)methyl)benzene* (**59**). White solid, 89% yield, ClogP 5.17, 146–147 °C. ^1^H NMR (400 MHz, CDCl_3_): *δ* 5.13 (*s*, 2H, OCH_2_), 7.03–7.05 (d, *J* = 6.8 Hz, 2H, 2 x ArH), 7.29 (*s*, 1H, ArH), 7.50–7.56 (*m*, 2H, 2 x ArH), 8.23–8.25 (d, *J* = 6.8 Hz, 2H, 2 x ArH); ^13 ^C NMR (100 MHz, CDCl_3_): *δ* 69.1 (OCH_2_), 114.8 (Ar), 124.8 (Ar), 126.0 (Ar), 126.6 (Ar), 129.3 (Ar), 130.8 (Ar), 132.6 (Ar), 133.1 (Ar), 135.7 (Ar), 142.0 (Ar), 163.1 (Ar). Anal. calcd for C_13_H_9_Cl_2_NO_3_: C, 52.38; H, 3.04; N, 4.70, found: C, 52.42; H, 3.06; N, 4.75.

*2-((4-nitrophenoxy)methyl)isoindoline-1,3-dione* (**60**). Yellow solid, 75% yield, ClogP 2.99, mp = 152–154 °C. [Lit. mp = 160–161 °C]. Characterisation data are in agreement with those reported in the literature^29^.

*2–(4-nitrophenoxy)-1–(3-nitrophenyl)ethan-1-one* (**61**). beige solid, 72% yield, ClogP 2.96, mp = 144–147 °C. ^1^H NMR (400 MHz, CDCl_3_): *δ* 5.46 (*s*, 2H, OCH_2_), 7.03–7.05 (d, *J* = 9.2 Hz, 2H, 2 x ArH), 7.77–7.81 (*t*, *J* = 8.0 Hz, 1H, ArH), 8.22–8.25 (d, *J* = 8.8 Hz, 2H, 2 × ArH), 8.35–8.37 (d, *J* = 7.6 Hz, 1H, ArH), 8.50–8.52 (d, *J* = 7.8 Hz, 1H, ArH), 8.85 (*s*, 1H, ArH); ^13 ^C NMR (100 MHz, CDCl_3_): *δ* 70.9 (OCH_2_), 114.8 (Ar), 123.1 (Ar), 126.1 (Ar), 128.5 (Ar), 130.4 (Ar), 133.8 (Ar), 135.2 (Ar), 142.5 (Ar), 148.6 (Ar), 162.4 (Ar), 191.4 (C = O). Anal. calcd for C_14_H_10_N_2_O_6_: C, 55.64; H, 3.34; N, 9.27, found: C, 55.78; H, 3.39; N, 9.33.

### 4.4. Cell lines and treatments

Human PC cell lines (AsPC-1, Capan-2 and BxPC-3) were cultured as previously described^5^. The three cell lines display different genetic profiles^5^. Human fibroblast cell line HFF-1 was purchased from American Type Culture Collection (ATCC; Manassas, VA, USA) and it was cultured in DMEM high glucose (4.5 g/L), supplemented with 15% FBS.

8-Hydroxy-5-nitroquinoline (nitroxoline) was purchased from Sigma (St. Louis, MO, USA). All compounds were dissolved in DMSO (stock solutions of 40 mM) and then in culture media for the final working concentrations. The final concentration of DMSO in the experiments was at most 0.27% and showed no cell toxicity.

### 4.5. Cell viability assay

Cell viability was evaluated by MTT assay (Sigma-Aldrich, St. Louis, MO, USA), essentially as previously described^30^. In particular, for initial screening all compounds were used at a fixed concentration of 40 μM for 48 h in the three PC cell lines. In concentration-response curves, MTT assays were carried out by incubating PC cell lines, or the normal fibroblast cell line with the most active molecules, at concentrations ranging from 0 μM to 54 μM for 48 h.

### 4.6. Clonogenic assay

Clonogenic assay was performed essentially as previously described (5). Capan-2 and BxPC-3 were seeded in 6-well plates at 10^3^ cells/well, while AsPC-1 were seeded in 6-well plates at 0.5 × 103 cells/well. After cell attachment, PC cell lines were treated for 48 h with compounds **33**, **40**, or with nitroxoline as indicated. Colonies were fixed when cells in the control vehicle formed colonies consisting of at least 30 cells^31^.

### 4.7. Half maximal inhibitory concentration (IC_50_) calculation and statistical analysis

IC_50_ values were calculated by CompuSyn software^32^. Comparisons of mean values were performed using an unpaired Student’s t-test or, for multiple comparisons, a one-way ANOVA followed by Dunnett’s test. A *p* values ≤0.05 was estimated as statistically significant.

## 5. Results and discussion

### 5.1. Antiproliferative effects and SAR studies

Starting from the results published on the anti-cancer potential of nitroxoline, our approach was oriented towards a wide exploration of the chemical spare around the phenolic moiety in the nitroxoline skeleton in order to attain newly synthesised derivatives with improved inhibitory activity on the viability of PC cell lines and with different chemical-physical characteristics to finely tune the predicted pharmacokinetic properties. Recent developments have started to address this issue^17^^,^^18^^,^^33^, but poor information remains, considering that those modifications could be important to better define the target of these compounds.

**Figure 3.**
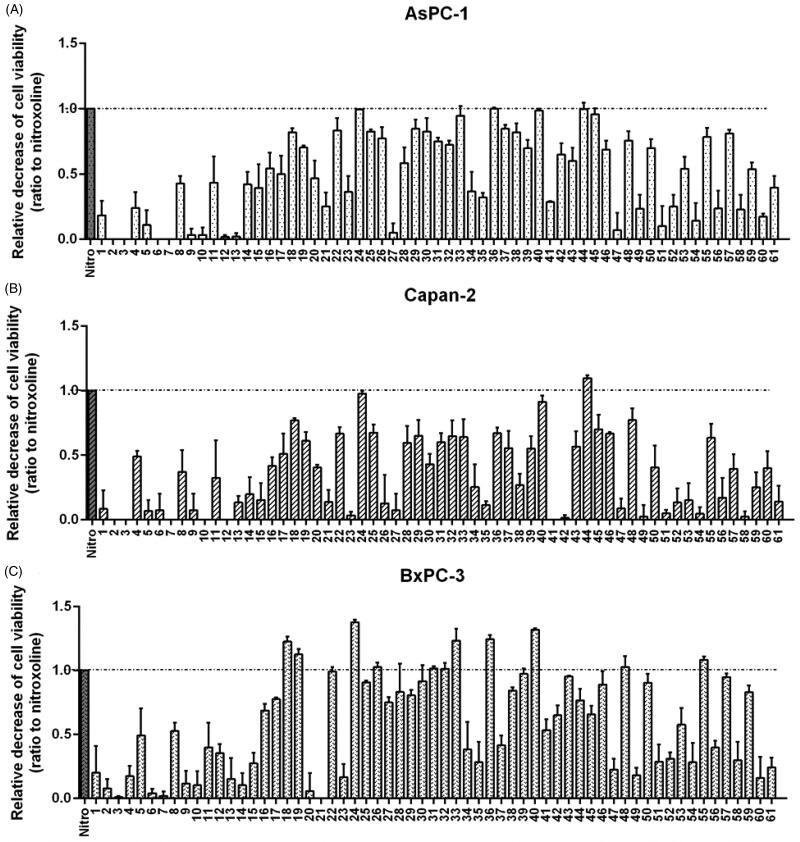
Screening of novel nitroxoline derivatives (1–61) on PC cell viability. Effects of novel derivatives on the viability of AsPC-1 (A), Capan-2 (B) and BxPC-3 (C) PC cell lines were assessed by MTT assays. The lead compound nitroxoline was included as a reference. MTT assays were performed by using compounds at 40 μM for 48 h and the histograms show the relative decrease of cell viability induced by treatments, as compared to nitroxoline. Data shown are the means ± SD of duplicate MTT experiments, each with quintuplicate determinations and are calculated as ratio relative to the lead compound nitroxoline (identified by a dashed line).

Based on the previously reported antiproliferative activity of nitroxoline on human PC cell lines (AsPC-1, Capan-2 and BxPC-3)^5^, we explored the effects of these novel compounds on cell viability in the same PC cells. To identify the most active compounds in the series, preliminary MTT experiments were performed by incubating the three PC cell lines with the 61 derivatives and with the lead compound nitroxoline, for 48 h at one-point screening concentration (40 μM) (Figure 3). Several novel derivatives inhibited viability more potently than nitroxoline and this effect was more evident in BXPC3 where compounds **18**, **19**, **24**, **33**, **36**, **40**, **48** and **55** were more effective than nitroxoline (Figure 3(C)). A similar potency was observed in Capan-2 with compound **44**, affecting a greater fraction of cells as compared to nitroxoline (Figure 3(B)). Notably, **24**, **33**, **36**, **40** and **44** were the most active compounds also in AsPC-1 cells (Figure 3(A)), although with a potency comparable to nitroxoline.

Structure-activity relationships (SARs) within this library suggested that the functionalization of the OH with aliphatic chains (**1–13**) led to a reduced inhibitory activity against the three cell lines with respect to nitroxoline disregarding the presence of double/triple bonds, functional groups, branched and long chains. Conversely, the insertion of a benzyl group (**11–40**) provided compounds of similar potency with respect to nitroxoline on AsPC-1 (**24**, **33**, **37**, **40**) and Capan-2 (**24**, **40**) cells. Preferred positions and substituents on the aryl ring were 3-Br, 4-CN, 3,5-diCH_3_ and 3,4-diCl. On BxPC-3 cells, these compounds displayed a more interesting scenario being some compounds (**22, 26, 31, 32, 39**) equipotent with respect to nitroxoline and compounds **18**, **19**, **24**, **33**, **36**, **40** even more potent at the same tested concentration. More in detail, only electron-withdrawing substituents (2-NO_2_, 2-Br, 3-Br, 4-CN, 2,6-diF, 3,4-diCl) on specific positions at the benzyl ring favoured the inhibitory activity. The presence of a carbonyl spacer between the OCH_2_ and the phenyl ring (**43–46**) furnished nitroxoline analogues with discrete effect on the three cell lines, whereas the molecular simplification to 4-nitro(thio)phenol derivatives (**47–61**) was detrimental for the inhibitory activity against AsPC-1 and Capan-2 cell lines. On BxPC-3 cells, compounds **48**, **50**, **55**, **57** and **59** were endowed with promising activity, despite the substitution of the oxygen with the sulphur led to a loss of biological activity (compare the couples **50**/**52** and **55**/**57**). Collectively, the deletion of the pyridine nucleus of the parent compound gave worse results. Thus, we selected the best-in-class compounds (**24**, **33**, **36**, **40** and **44)** for further characterisation of their chemical-physical properties and antiproliferative effects.

To evaluate the concentration-dependent effects of the most promising compounds, PC cell lines were treated with **24**, **33**, **36**, **40** and **44** for 48 h at concentrations of 2, 6, 18 and 54 μM, or with vehicle (control) (Figure S1). Selected compounds affected PC cell viability in a concentration-dependent manner (Figure S1). IC_50_ values were calculated by CompuSyn software (Table 1). Compounds **33** and **40** had the lowest IC_50_ values as compared to other derivatives and to nitroxoline, displaying the most consistent antiproliferative effects across the three PC cell lines. Interestingly, in AsPC-1 and Capan-2 compounds **33** and **40** had IC_50_ values lower than those previously obtained with erlotinib^5^, a targeted agent approved for PC treatment. Moreover, in BxPC-3, the least sensitive PC cell line to nitroxoline, but sensitive to erlotinib, the IC_50_ of compound **40** was lower as compared to both agents^5^.

**Table 1.** IC_50_ values for compounds **24**, **33**, **36**, **40**, **44** and nitroxoline in PC cell lines.

Compound	IC_50_ (µM)		
	AsPC-1	Capan-2	BxPC-3
Nitroxoline*	26.8	16.9	41.2
**24**	26.1	40.2	47.1
**33**	13.7	17.8	20.7
**36**	17.7	21.6	24.5
**40**	4.9	9.8	9.7
**44**	20.1	27.1	39.5
Erlotinib*	22.8	30.5	10.9

* IC_50_ values for Nitroxoline and Erlotinib were previously reported by us^5^.

**Figure 4.**
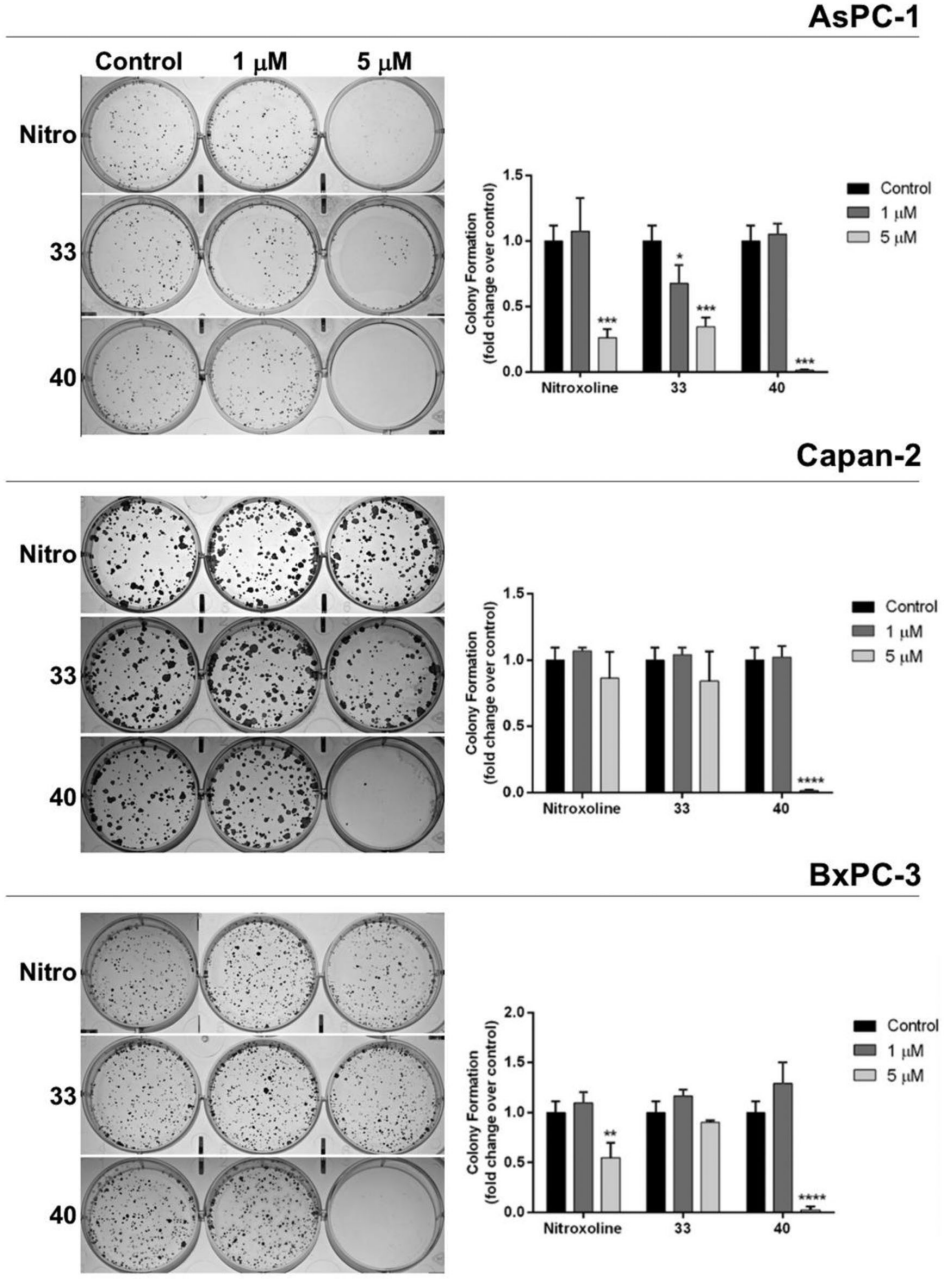
Effect of compounds **33**, **40** and nitroxoline on the clonogenic capacity of AsPC-1, Capan-2 and BxPC-3 PC cell lines. Representative plates of colony formation assays for the three PC cell lines exposed to tested compounds at 1 μM, 5 μM, or vehicle (control) are shown. Data shown in the histograms are the means ± SD of two independent experiments and are expressed as fold change relative to control (*p < 0.05; **p < 0.01; ***p < 0.001; ****p < 0.0001).

The two compounds with the lowest IC_50_ values in MTT assays were also tested for their ability to interfere with clonogenicity in PC cells. In particular, we compared the effects of **33**, **40** and nitroxoline on colony formation ability of the three PC cell lines (Figure 4). At 1 μM only compound **33** had a significant effect, reducing clonogenicity of AsPC-1. Conversely, when used at 5 μM the effects of the three compounds on clonogenicity appeared more consistent. In particular compound **33** markedly reduced clonogenicity of AsPC-1, nitroxoline affected both AsPC-1 and BxPC3, and compound **40** dramatically reduced clonogenicity of the three PC cell lines, consistently showing a greater effect as compared to **33** and nitroxoline used at the same concentrations. Remarkably, the effects of these novel compounds on cell self-renewal capacity in PC cells were stronger than those that we previously reported with erlotinib at higher concentrations^5^.

We next tested the effects of the most active compound on viability of normal HFF-1 fibroblast cells. Notably, compound **40** showed low toxicity against normal HFF-1 cells (IC_50_ 23.1 μM) and selectivity index (SI) values comparable or superior to nitroxoline in the three PC cell lines (Table 2 and Figure S2). In particular, compound **40** has similar selectivity as nitroxoline in Capan-2, but is much more selective in AsPC-1 and BxPC-3, suggesting that compound **40** may be more effective and safer than nitroxoline.

**Table 2.** Selectivity index (SI) values for compound **40** and nitroxoline.

Compound	Selectivity index (SI)		
	AsPC-1	Capan-2	BxPC-3
**40**	4.71	2.36	2.38
Nitroxoline	1.69	2.69	1.10

SI values are calculated for each compound as follows: SI = IC_50_ on normal fibroblast cells (HFF-1)/IC_50_ on cancer cell line. IC_50_ value of nitroxoline on HFF-1 is 45.4 μM (Figure S2).

### 5.2. *In silico* pharmacokinetic parameters and target prediction

In Table 3, the physical–chemical, pharmacokinetics and medicinal chemistry parameters obtained from the commercially available web tool SwissADME^23^ are reported, aiming to describe some properties of the tested compounds that are fundamental for a proper drug development. On the basis of the well-known Lipinski’s *rule of five* is possible to evaluate the drug-likeness of one compound, i.e., the probability that the molecule will be effective as an oral drug. As one can see, all the evaluated compounds fully comply with the limits of the Lipinski’s rule, supporting their feasible oral use (Lipinski violation is equal 0 for all the analogues), similarly to the lead compound nitroxoline.

**Table 3.** *In silico* evaluated physicochemical properties of nitroxoline and the most potent compounds **24**, **33**, **36**, **40**, and **44**.

Compound	Nitroxoline	**24**	**33**	**36**	**40**	**44**
Molecular weight (MW)	190.16	359.17	305.29	316.26	349.17	353.29
H-bond acceptors (HBA)	4	4	5	6	4	7
H-bond donators (HBD)	1	0	0	0	0	0
Consensus Log P*	1.17	3.22	2.37	3.21	3.67	1.83
Lipinski violations	0	0	0	0	0	0
GI absorption	High	High	High	High	High	High
P-gp substrate	No	No	No	No	No	No
PAINS alerts	0	0	0	0	0	0

* Arithmetic mean of the values predicted by five *in silico* methods: XLOGP3, WLOGP, MLOGP, SILICOS-IT, iLOGP. Parameters range required to satisfy the Lipinski’s rule of five: MW ≤ 500 g/mol, HBD ≤ 5, HBA ≤ 10, log *P* ≤ 5.

The capability of these compounds to be passively adsorbed in the gastrointestinal tract is an important information influencing their bioavailability after oral administration; even in this case all the examined compounds exhibited promising results. One of the Achilles’ heels of some antitumor drugs is the propensity to be substrate of the permeability glycoprotein (P-gp). This protein promotes the energy-dependent efflux of cytotoxic drugs out of the cytosol and its overexpression in tumour cells leads to multi-drug resistance (MDS), which reduces the efficacy of antitumor treatments. The outcomes extrapolated by *in silico* studies rule out the possibility that these compounds could be substrate of P-gp, reinforcing their role as antitumor drugs. Finally, the presence of PAINS (Pan Assay Interference Compounds) that are substructures able to elicit promiscuous pharmacological behaviour, was also evaluated showing as all the compounds are devoid of this attribute.

**Figure 5.**
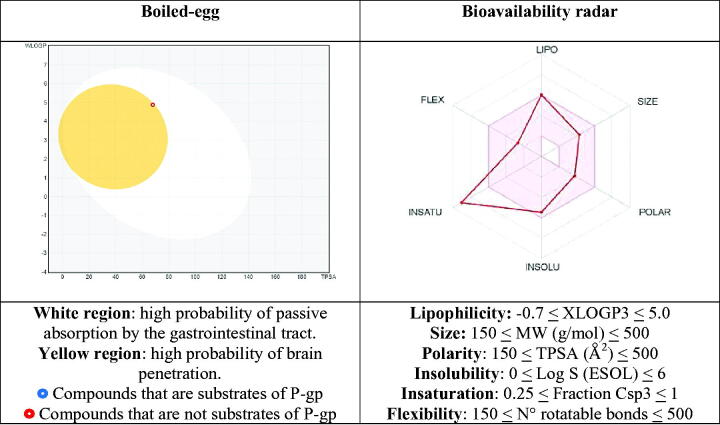
Representation of the boiled-egg graph and bioavailability radar calculated by SwissADME web-tool.

For the most active analogue (**40**), we also reported (Figure 5) the boiled-egg and bioavailability radar pictures, the two graphical outputs of the SwissADME tool. These graphs are based on the data reported in Table 4. The boiled-egg graph is obtained by considering the two parameters WLOGP, as a lipophilic index, and TPSA, as a measure of apparent polarity, enriched with the information about the risk to be substrate of P-gp. It allows, at first glance, to predict simultaneously two ADME parameters that are the passive absorption at the gastrointestinal tract (white area) and the ability to permeate the blood brain barrier (BBB, yellow area), along with the susceptibility to P-gp depending on the colour of the dot (the red dot means that the compound is not a substrate of the P-gp; on the contrary, the blue dot indicates a putative substrate of the P-gp). As one can see, the most active compound **40** is contained in the yellow area (even if it is to the limit) and is pictured with a red dot, signifying that it is likely able to cross the BBB, it is passively absorbed at the GI level and is not effluxed by P-gp.

**Table 4.** *In silico* estimated physicochemical parameters of compound **40**, used to device the boiled-egg graph and bioavailability radar.

Cmpd	WLOGP^a^	TPSA (Å^2^)^a^,^b^	XLOGP3^b^	Log S (ESOL)^b^	MW^b^	Csp3^b^	N° of rotatable bonds^b^
**40**	4.88	67.94	5.07	−5.45	349.17	0.06	4

^a^ Parameters used for the boiled-egg graph.

^b^ Parameters used for the bioavailability radar. Bioavailability radar parameters functional ranges: XLOGP3 between −0.7 and +5.0, MW between 150 and 500 g/mol, TPSA between 20 and 130Å^2^, log S not higher than 6, saturation: fraction of carbons in the sp^3^ hybridisation not less than 0.25, and flexibility: no more than 9 rotatable bonds.

Furthermore, the bioavailability radar provides the drug-likeness representation of the compound. The pink area is indicative for the optimal range of each physicochemical property (lipophilicity, size, polarity, solubility, saturation, flexibility) required to be bioavailable after oral administration. For the analogue **40** only the insaturation parameter is out of the desired range, while all the others fit into the red-depicted area, accounting for a putative moderate oral bioavailability.

With the aim to identify the putative target/s of our compounds we also exploited the web-tool SwissTargetPrediction^24^, performing the analysis on the lead nitroxoline and compound **40**. In Tables 5 and 6, we reported the data concerning the most probable targets found by the web-tool, that is, the ones exhibiting the highest values of probability to be a target. Remarkably, among targets with the highest probability score, cyclooxygenase-2 was previously unknown to be a target of nitroxoline, while Type-2 methionine aminopeptidase (METAP2) activity was previously reported to be inhibited by nitroxoline^12^. Moreover, among the molecules with a lower probability score PARP1 is a known target of nitroxoline, which induces relevant effects on PARP cleavage^5^. On the contrary, for compound **40** we did not obtain a main preference for one protein; thus, further and deep studies could shed light on its putative targets.

**Table 5.** Protein target prediction for nitroxoline.

Target	Common name	Uniprot ID	ChEMBL ID	Target Class	Probability
Cyclooxygenase-2	PTGS2	P35354	CHEMBL230	Oxidoreductase	1
Methionine aminopeptidase 2	METAP2	P50579	CHEMBL3922	Protease	1
DNA excision repair protein ERCC-5	ERCC5	P28715	CHEMBL4736	Other nuclear protein	0.03123
Ribonuclease H1	RNASEH1	O60930	CHEMBL5893	Hydrolase	0.03123
Poly [ADP-ribose] polymerase-1	PARP1	P09874	CHEMBL3105	Transferase	0.03123
Proteasome Macropain subunit MB1	PSMB5	P28074	CHEMBL4662	Protease	0.03123
Indoleamine 2,3-dioxygenase	IDO1	P14902	CHEMBL4685	Oxidoreductase	0.03123
Tryptophan 2,3-dioxygenase (by homology)	TDO2	P48775	CHEMBL2140	Oxidoreductase	0.03123

**Table 6.** Protein target prediction for compound **40**.

Target	Common name	Uniprot ID	ChEMBL ID	Target Class	Probability
Cyclooxygenase-1 (by homology)	PTGS1	P23219	CHEMBL221	Oxidoreductase	0.10161
TGF-beta receptor type I	TGFBR1	P36897	CHEMBL4439	Kinase	0.10161
Macrophage migration inhibitory factor	MIF	P14174	CHEMBL2085	Isomerase	0.10161
Carbonic anhydrase XII	CA12	O43570	CHEMBL3242	Lyase	0.10161
Phosphodiesterase 5A	PDE5A	O76074	CHEMBL1827	Phosphodiesterase	0.10161
ATP-binding cassette sub-family G member 2	ABCG2	Q9UNQ0	CHEMBL5393	Primary active transporter	0.10161
Arachidonate 15-lipoxygenase	ALOX15	P16050	CHEMBL2903	Oxidoreductase	0.10161
Phosphatidylinositol-5-phosphate 4-kinase type-2 gamma	PIP4K2C	Q8TBX8	CHEMBL1770034	Kinase	0.10161
DNA topoisomerase II alpha	TOP2A	P11388	CHEMBL1806	Isomerase	0.10161
Vascular cell adhesion protein 1	VCAM1	P19320	CHEMBL3735	Adhesion	0.10161

## 6. Conclusion

Our study explored the impact of the chemical modifications of the core nucleus of a well-known antibiotic, nitroxoline, that recently emerged as a promising candidate for its antiproliferative effects on PC cell lines. The functionalization of the phenolic OH with substituted benzyl rings led to an improvement of the biological activity with respect to the parent compound and erlotinib, whereas the introduction of alkyl groups, the deletion of the pyridine ring or the bioisosteric change from oxygen to sulphur were detrimental for the reported activity. Moreover, the most promising compounds were further evaluated in terms of clonogenicity reduction, *in silico* drug-likeness and cytotoxicity on normal human fibroblasts. Collectively, our data supported the exploration of this chemical scaffold to propose new compounds with alternative mechanisms of action for the treatment of pancreatic cancer.

## Supplementary Material

Supplemental Material
