# Supplementary material for: Synthesis and evaluation of a large library of nitroxoline derivatives as pancreatic cancer antiproliferative agents
Source: J Enzyme Inhib Med Chem. 2020 Jun 26;35(1):1331–44. doi: 10.1080/14756366.2020.1780228 (PMC7470072; doi:10.1080/14756366.2020.1780228)

Synthesis and evaluation of a large library of nitroxoline derivatives as pancreatic cancer antiproliferative agents

Serena Veschi[a], Simone Carradori[a,*], Laura De Lellis[a], Rosalba Florio[a], Davide Brocco[a], Daniela Secci[b], Paolo Guglielmi[b], Mattia Spano[b], Anatoly P. Sobolev[c], Alessandro Cama[a]

[a]Department of Pharmacy, "G. d'Annunzio" University of Chieti-Pescara, Chieti 66100, Italy

[b]Dipartimento di Chimica e Tecnologie del Farmaco, Sapienza Università di Roma, Rome 00185, Italy

[c]Istituto per i Sistemi Biologici, Laboratorio di Risonanza Magnetica "Segre-Capitani", CNR, Via Salaria Km 29.300, 00015 Monterotondo (Rome), Italy

*Corresponding author: Simone Carradori PhD, Department of Pharmacy, "G. d'Annunzio" University of Chieti-Pescara, Chieti 66100, Italy; Tel. +39 0871 3554583; e-mail: simone.carradori@unich.it

Figure S1. Concentration-response curves of compounds 24, 33, 36, 40 and 44 on viability of PC cell lines. We analyzed by MTT the effect of compounds **24**, **33**, **36**, **40** and **44** on the viability of AsPC-1, Capan-2 and BxPC-3 treated for 48 hours at concentrations ranging from 0 (vehicle, control) to 54 μM. The compounds affected cell viability in a dose-dependent manner, with distinct sensitivities for the three PC cell lines. Data shown are the means +SD of two MTT assays with quintuplicate determinations. Statistically significant differences between control and each compound concentration were expressed as *p<0.05; **p<0.01; ***p<0.001; ****p<0.0001.

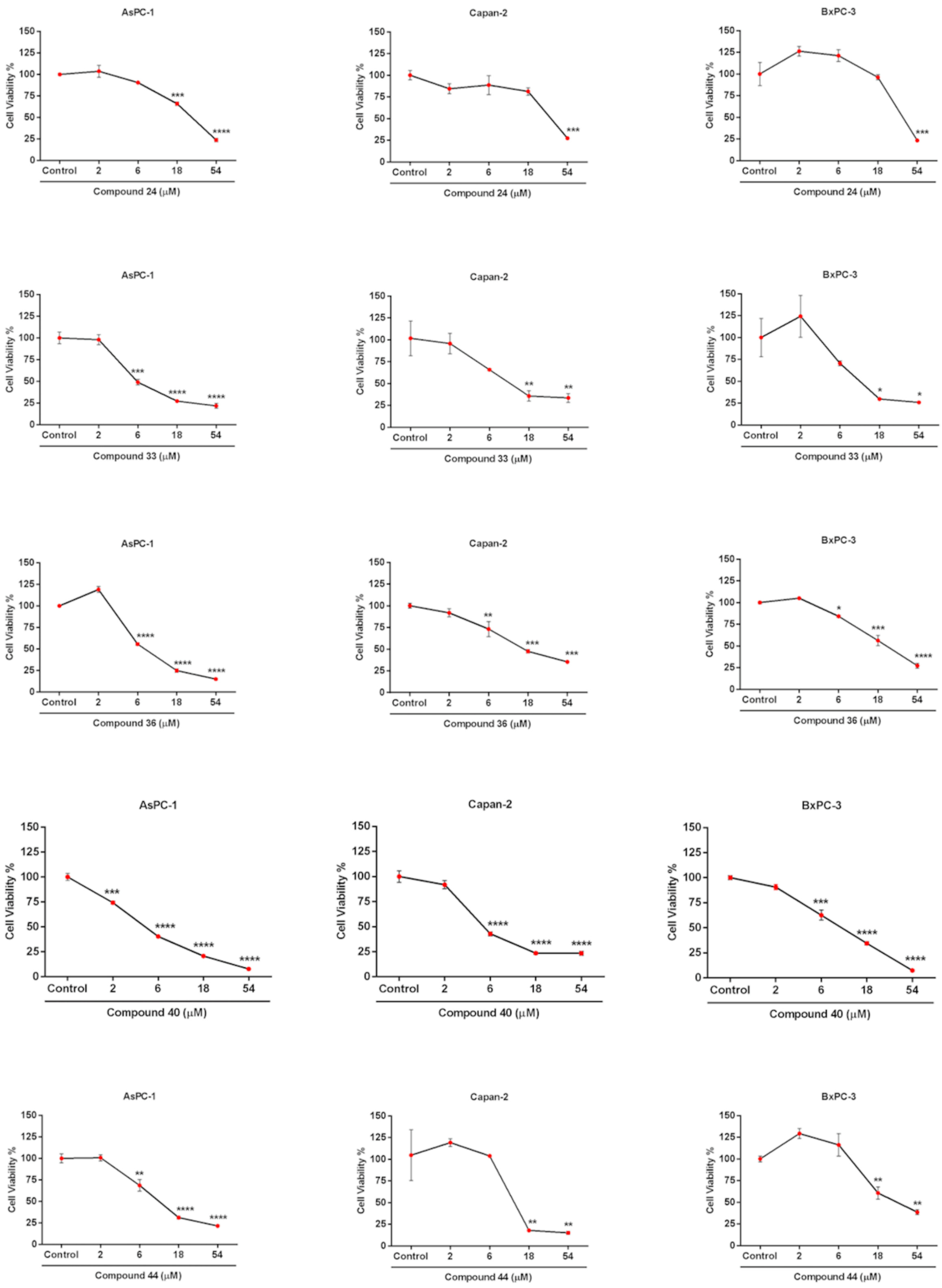

Figure S2. Concentration-response curves of nitroxoline and compounds 40 on viability of normal HFF-1 fibroblast cells. We analyzed by MTT the effect of nitroxoline and compound **40** on viability of HFF-1 treated for 48 hours at concentrations ranging from 0 (vehicle, control) to 54 µM. IC_{50} values of both nitroxoline and compound **40** were higher than those obtained in PC cancer cell lines (Table 1). IC_{50} values were calculated by CompuSyn software. Data shown are the means ±SD of two MTT assays with quintuplicate determinations. Statistically significant differences between control and each compound concentration were expressed as $^{**}p<0.01$; $^{***}p<0.001$; $^{****}p<0.0001$.

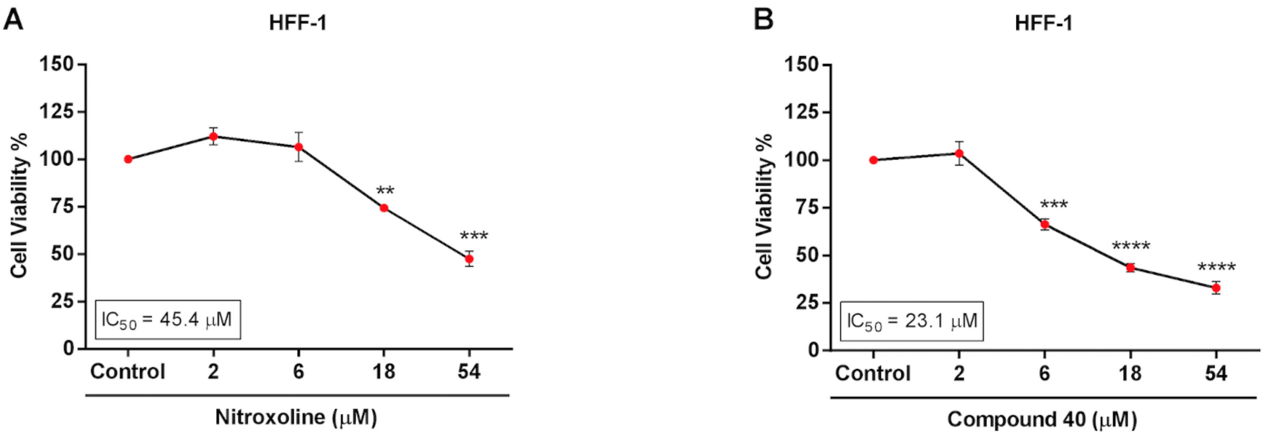

Supplement: Supplemental Material [file IENZ_A_1780228_SM4090.pdf]
